# The effect of realistic geometries on the susceptibility‐weighted MR signal in white matter

**DOI:** 10.1002/mrm.26689

**Published:** 2017-04-10

**Authors:** Tianyou Xu, Sean Foxley, Michiel Kleinnijenhuis, Way Cherng Chen, Karla L. Miller

**Affiliations:** ^1^ Oxford Centre for Functional MRI of the Brain University of Oxford Oxford United Kingdom; ^2^ Singapore Bioimaging Consortium, A*STAR Singapore

**Keywords:** R2*, GRE phase signal, magnetic susceptibility modeling, white matter microstructure, myelin

## Abstract

**Purpose:**

To investigate the effect of realistic microstructural geometry on the susceptibility‐weighted MR signal in white matter (WM), with application to demyelination.

**Methods:**

Previous work has modeled susceptibility‐weighted signals under the assumption that axons are cylindrical. In this study, we explored the implications of this assumption by considering the effect of more realistic geometries. A three‐compartment WM model incorporating relevant properties based on the literature was used to predict the MR signal. Myelinated axons were modeled with several cross‐sectional geometries of increasing realism: nested circles, warped/elliptical circles, and measured axonal geometries from electron micrographs. Signal simulations from the different microstructural geometries were compared with measured signals from a cuprizone mouse model with varying degrees of demyelination.

**Results:**

Simulation results suggest that axonal geometry affects the MR signal. Predictions with realistic models were significantly different compared with circular models under the same microstructural tissue properties, for simulations with and without diffusion.

**Conclusion:**

The geometry of axons affects the MR signal significantly. Literature estimates of myelin susceptibility, which are based on fitting biophysical models to the MR signal, are likely to be biased by the assumed geometry, as will any derived microstructural properties. Magn Reson Med 79:489–500, 2018. © 2017 International Society for Magnetic Resonance in Medicine.

## INTRODUCTION

Myelin microstructure in white matter (WM) is important for healthy brain function and in neurological disease. In human and animal brains, normal myelin formation supports healthy development and promotes vital processes such as neuroplasticity 1. In contrast, abnormal myelin conditions, such as demyelination, are associated with many forms of neuropathology such as multiple sclerosis 2. Given myelin's important role in brain function, a long‐standing goal in human neuroscience has been to noninvasively estimate properties of myelin—its volume fraction in WM, or more specifically, intact myelin volume fraction—from the MR signal.

There are a number of MR‐based markers of myelin, including multicompartment T_1_, $T_{2}^{*}$, and magnetization transfer mapping 3, 4, 5, 6. In addition, myelin has a magnetic susceptibility *χ* that is offset to its environment. This arises from myelin's unique chemical composition and ordering of phospholipids within the myelin sheath structure. Following empirical works demonstrating that the frequency dependent MR signal (e.g., spectroscopic imaging) may reflect localized differences in magnetic susceptibility ***χ*** 7, 8, 9, recent studies have shown that the magnetic susceptibility of myelin strongly influences the gradient echo (GRE) signal, including both signal phase and magnitude 10, 11, 12, 13, 14.

Several biophysical models of WM based on myelin microstructure have been used to interpret the measured GRE signal. Factors influencing this signal include relative volume fractions of myelin and intra‐/extra‐axonal water, g‐ratio (thickness of the myelin sheath), magnetization exchange with myelin water, the presence of paramagnetic iron and the magnetic susceptibility of myelin 4, 15, 16, 17, 18. Moreover, there is recent evidence that myelin exhibits susceptibility anisotropy, where the magnetic susceptibility depends on the orientation of the phospholipids in myelin with respect to the magnetic field, B_0_ 4, 11, 14, 19, 20, 21.

The present study focuses on the specific geometry of the myelinated axon and its effect upon the susceptibility‐weighted signal. Existing models use nested cylinders to describe axons, assuming circular geometries 4, 14, 15. In reality, a diversity of axonal shapes and myelin geometries exist in WM. While simulations using circular shapes benefit from simplicity, the effects of this assumption have not been studied. Given the role that shape has in altering the field perturbations caused by susceptibility‐shifted structures, shape is a potential confound in the extraction of microstructure parameters (e.g., myelin thickness).

Myelinated axons perpendicular to the main magnetic field were modeled in two dimensions with several variations. First, we modeled single axons and axon bundles using circular geometries. Next, we modeled the role of myelin shape on the MR signal by distorting circular geometries. We considered more realistic geometries by using a structural template of myelin microstructure derived from electron microscopy (EM) data. Finally, the signal predictions of circular and EM‐based geometries were evaluated against data acquired in a mouse model of demyelination.

## THEORY

**Figure 1:**
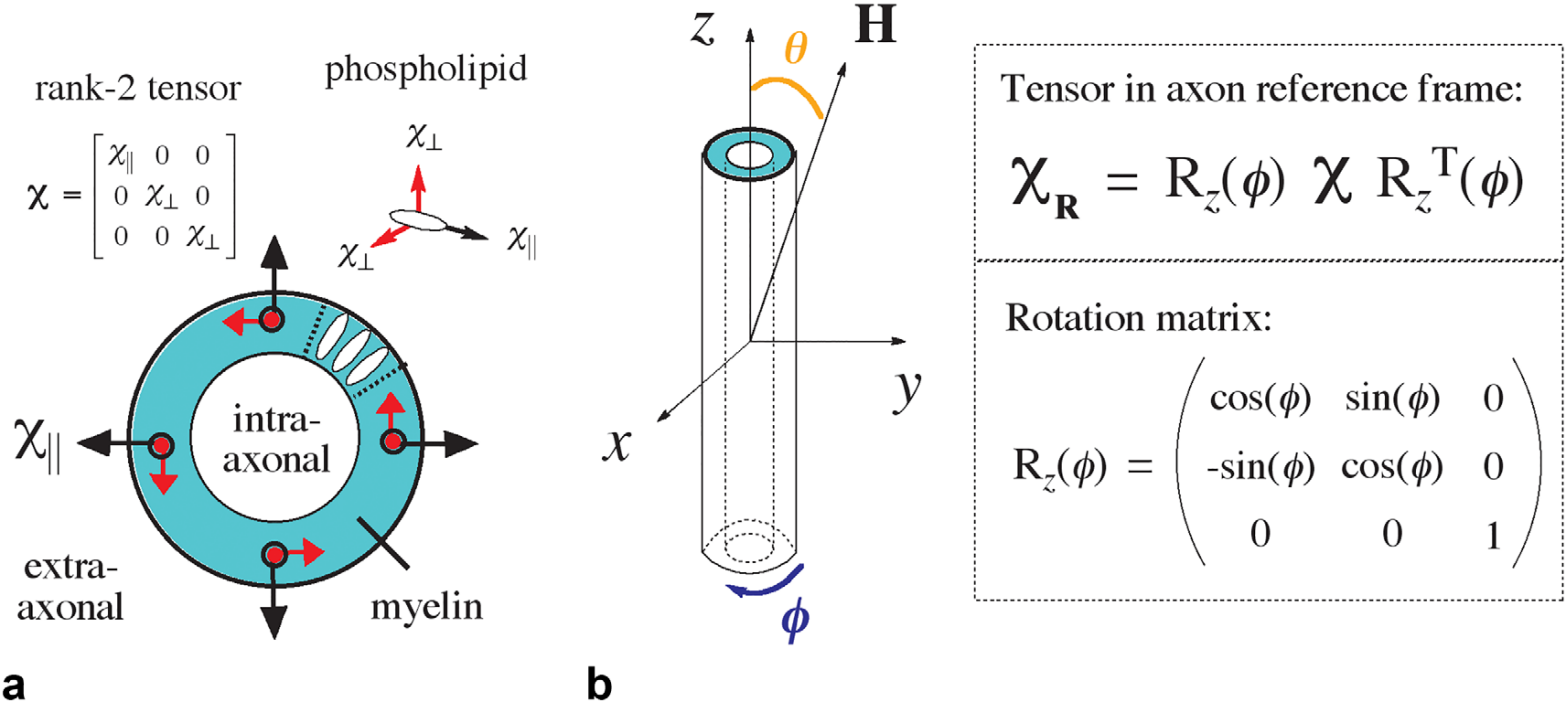
(**a**) The magnetic susceptibility anisotropy of myelin is suggested to originate from its constituent phospholipid bilayer unit, which is a radially oriented in the myelin sheath. Susceptibility anisotropy is described mathematically as a rank‐2 tensor. (**b**) Assuming the longitudinal component of the tensor *χ*_||_ is aligned with *x*, a rotation matrix about *z* is applied to transform the tensor into the common frame of the axon. Spherical coordinates are used where *ϕ* is the azimuthal angle and *θ* is elevation.

Previous biophysical models of axons have assumed idealized packings of nested cylinders that are parallel and infinite along one direction and circular in the orthogonal plane 15. These geometric models have been used to create maps of relative magnetic susceptibility that are then used to forward calculate the corresponding local field perturbations 22, 23. For isotropic magnetic susceptibility, the field perturbations are generated through pointwise multiplication of a dipole kernel with the susceptibility map in Fourier space, followed by an inverse Fourier transform. This calculation becomes more complicated under magnetic susceptibility anisotropy 4, 11, 14, which has been suggested to originate from the radial stacking/orientation of the phospholipid bilayers comprising the myelin sheath (Fig. 1a) 24, 25, 26. A tensor formulation of the Fourier method is used to incorporate susceptibility anisotropy in the calculation of the microstructural field 14, 21.

**Figure 2:**
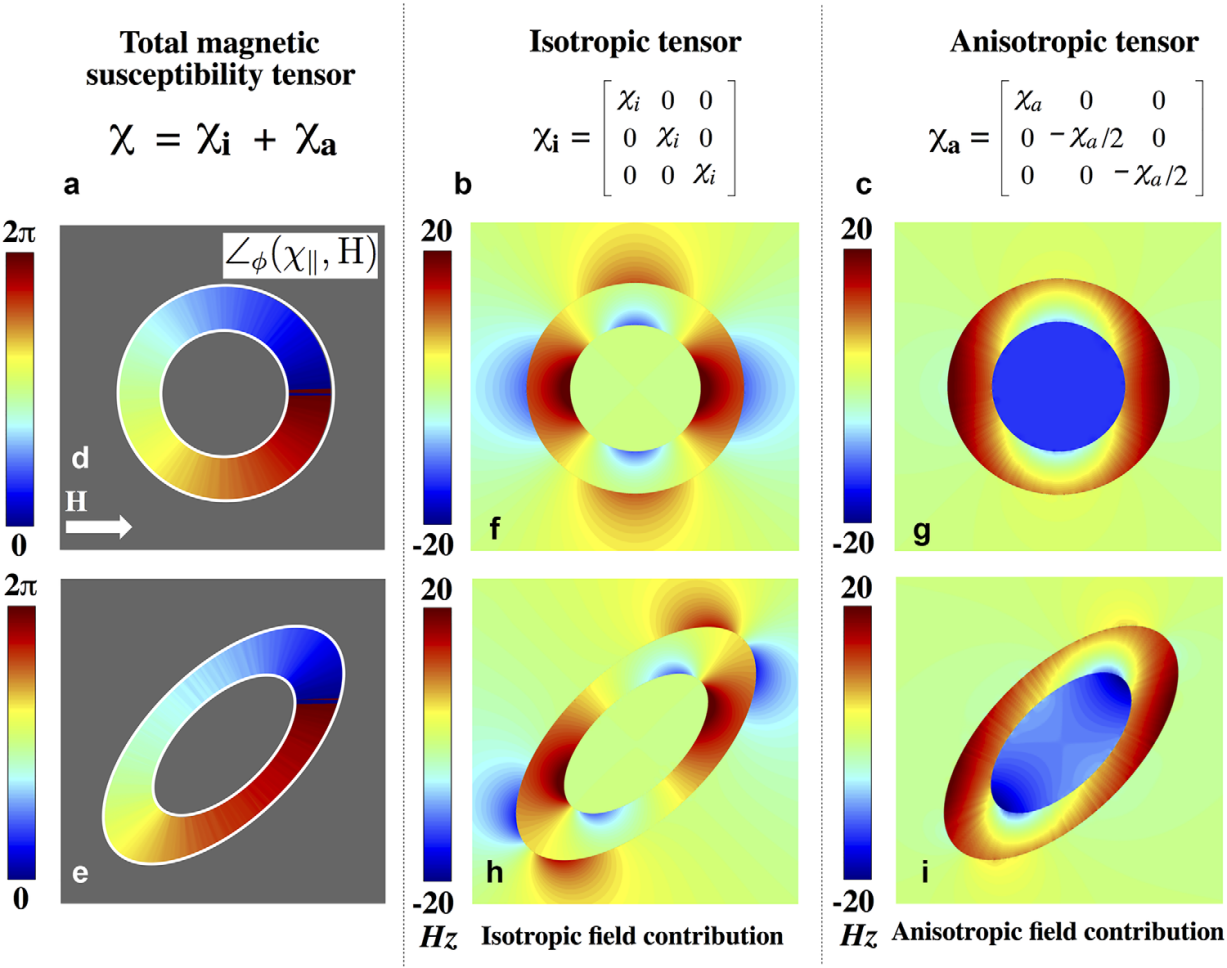
(**a**) The total magnetic susceptibility of myelin can be expressed as the summation of two components: an isotropic component, *χ*_i_ and the anisotropic component, *χ*_a_. (**b, c**) Tensor formulations for *χ*_i_ and *χ*_a_ in the unrotated frame. (**d, e**) Orientations of the longitudinal tensor component *χ*_||_ with **H** is plotted about the azimuth (∠*ϕ*) for two geometric cases: a nested cylinder and a nested elliptical fiber model. Longitudinal axes of the fibers are assumed to be orthogonal to **H** (∠*θ* **=** π/2); perpendicular cross‐sections are shown. (**f**–**i**) Isotropic and anisotropic fields corresponding to the nested cylinder (f, g) and elliptical geometries (h, i).

In this study, the intra‐/extra‐axonal regions are considered as an implicit reference (zero susceptibility) from which the susceptibility of myelin is offset. The total magnetic susceptibility of myelin can be expressed as the summation of isotropic and anisotropic susceptibility components defined by rank‐2 tensors (Fig. 2). Directional susceptibility anisotropy, characteristic of the phospholipid bilayer, is described by a tensor with nonequivalent diagonal components where *χ*_∥_ ≠ *χ*_⊥_ (Fig. 1a) 14. *χ* is transformed into the common reference frame of the axon by a rotation matrix (Fig. 1b). Next, the spatial susceptibility‐tensor map is used to forward calculate the corresponding field perturbation using the Fourier expression in the following equation 21:

(1) $$\Delta f\left(r,\phi \right)=FT^{-1}\left\{\frac{1}{3}{\overset{⁁}{H}}^{T}FT\left\{\chi_{R}\left(r,\phi \right)\right\}\overset{⁁}{H}-{\overset{⁁}{H}}^{T}k\frac{k^{T}FT\left\{\chi_{R}\left(r,\phi \right)\right\}\overset{⁁}{H}}{k^{2}}\right\}\bar{\gamma }H$$

where Δf(r,ϕ) is the off‐resonance frequency in *Hz*, χ_R_(*r,ϕ*) represents the spatial susceptibility tensor map defined in the reference frame of the axon (Fig. 1b), **H** = [sin(*θ*), 0, cos(*θ*)]*H*_*0*_ is the applied magnetic field, *θ* is the orientation of the fiber to the magnetic field, **k** = [*k_x_, k_y_, k_z_*] is the spatial frequency vector, $\bar{\gamma }$ is the gyromagnetic ratio, and *FT* is the Fourier Transform. The applied magnetic field **H** in Equation [1] is equivalent to B_0_ of the MRI magnet. Details on the field perturbation calculations are provided in the Supporting Information.

## METHODS

### Microstructure Model

We modeled several geometries, including idealized cylinders with circular cross‐sections, elliptic cylinders with elliptical cross‐sections, and hyperrealistic geometries based on EM.

**Figure 3:**
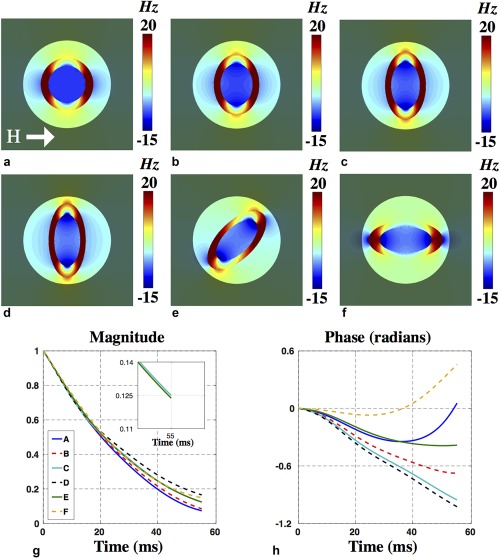
(**a**–**c**) Field perturbations of elliptical geometries of increasing eccentricity starting from 0 or a circle. (**d**–**f**) The effect of in‐plane rotations about the applied field. (**g, h**) The simulated signal magnitude and phase corresponding to panels a–f.

For a proof‐of‐principle examination of the role of shape, we performed field perturbations corresponding to a single axon with elliptical cross section and increasing eccentricities, 0, 0.66, 0.80, and 0.87, corresponding to minor‐to‐major axis ratios of 1, 4/3, 5/3, and 2, respectively. Changes to eccentricity were made without change to myelin or intra‐/extra‐axonal areas to conserve g‐ratio, which was set to 0.7 27. In circular axons, the g‐ratio is the ratio of the inner radius to the outer radius, while for noncircular axons $g=\sqrt{A_{i}/A_{t}}$ with intra‐axonal and total axonal areas $A_{i}$ and *A*_*t*_. The effect of rotation on the ellipse with respect to **H** is also examined (Fig. 3e,f). Simulations were performed on a 500 × 500 array, spanning 3 × 3 μm^2^, with **H** defined orthogonal to the longitudinal axis of the fiber.

**Figure 4:**
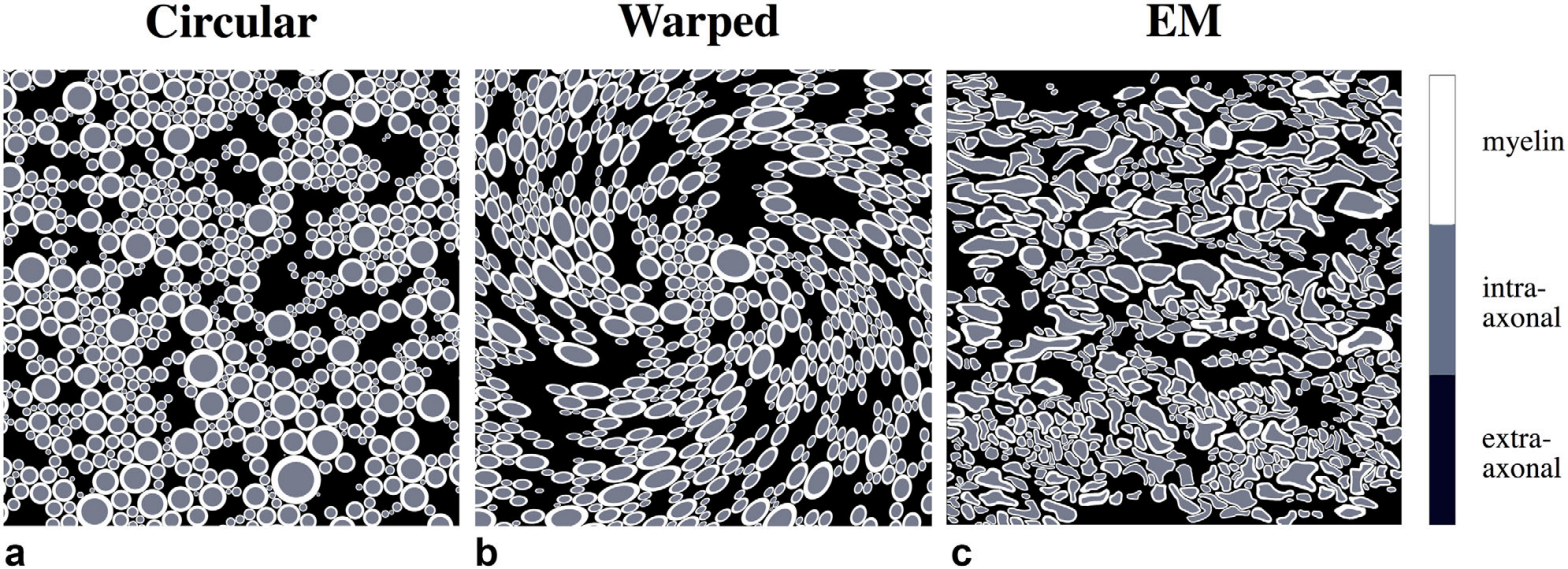
Three models of increasing geometric realism. (a) Circular axons (n *=* 1434). (b) Elliptical axons (warped circles; n *=* 1434). (c) Axons segmented from EM data of mouse WM (n *=* 602). The circular and warped geometries were designed to match relevant properties of the EM segmentation: fiber density and myelin thickness. Myelin structure is shown in white, intra‐axonal space is shown in gray, and extra‐axonal space is shown in black.

Packed axons were generated on a 4454 × 4454 array, spanning 37 × 37 μm^2^ by random close packing of circles (n *=* 1434). Circle/axon sizes followed a Gamma distribution (α = 5.7) about a mean radius of 0.46 μm to match EM data (n *=* 602 axons). The packing algorithm was developed in‐house and is described in the Supporting Information. Results of the packing are shown in Supporting Figure S1. The packed axons were also warped to create a second geometry of packed ellipses using an algorithm package, *twirl1.m* 28 (parameters: a = 1.5; b = 0.8; c = 2.0; d = 1/400). This warping transformation conserves the g‐ratio and fiber density (Fig. 4a,b).

To model susceptibility anisotropy, it is necessary to determine the angle at which phospholipids in the myelin sheath would be oriented with respect to **H**. This was achieved by segmenting the myelin structure into azimuthally stacked, rectangular quadrilaterals. Further details are described in Supporting Information (Supporting Fig. S2).

Demyelination was modeled for cylindrical and EM‐based geometries by thinning the myelin structure from the inside out, producing a range of g‐ratios from 0.70 (normal) to 0.98 (significant loss of myelin). Further details regarding demyelination simulations are provided in the Supporting Information.

### Diffusion Model

Diffusion within axonal field perturbations affects the susceptibility‐weighted signal for longer echo times (>20 ms) 4 and was simulated for both EM and circular geometries. Monte Carlo with 100,000 spins were conducted in two dimensions, given that field perturbations are constant in the third direction (along axons). We chose a step time corresponding to 4 pixels in the model geometry (small relative to the space between axons) and the number of steps corresponding to 55 ms (EM model: step time 0.0001 ms, 550,000 steps; circular model: step time 0.0001612 ms, 341,191 steps). In the non‐myelin compartments, the diffusion coefficient was set to match measurements along axons (D = 2 μm^2^/ms), representing diffusion in the absence of axonal hindrance 29. Diffusion was assumed to be negligible for the myelin compartment 30. The effect of diffusion on each spin is calculated by summing the phase accrual experienced with each time step. To demonstrate the effect of diffusion on modelling, we also present some results without static magnetization (D = 0 μm^2^/ms). Details regarding the diffusion simulations, model validation, and diffusion‐weighted signal calculation are provided in Supporting Figure S3.

### Signal Calculation

**Figure 5:**
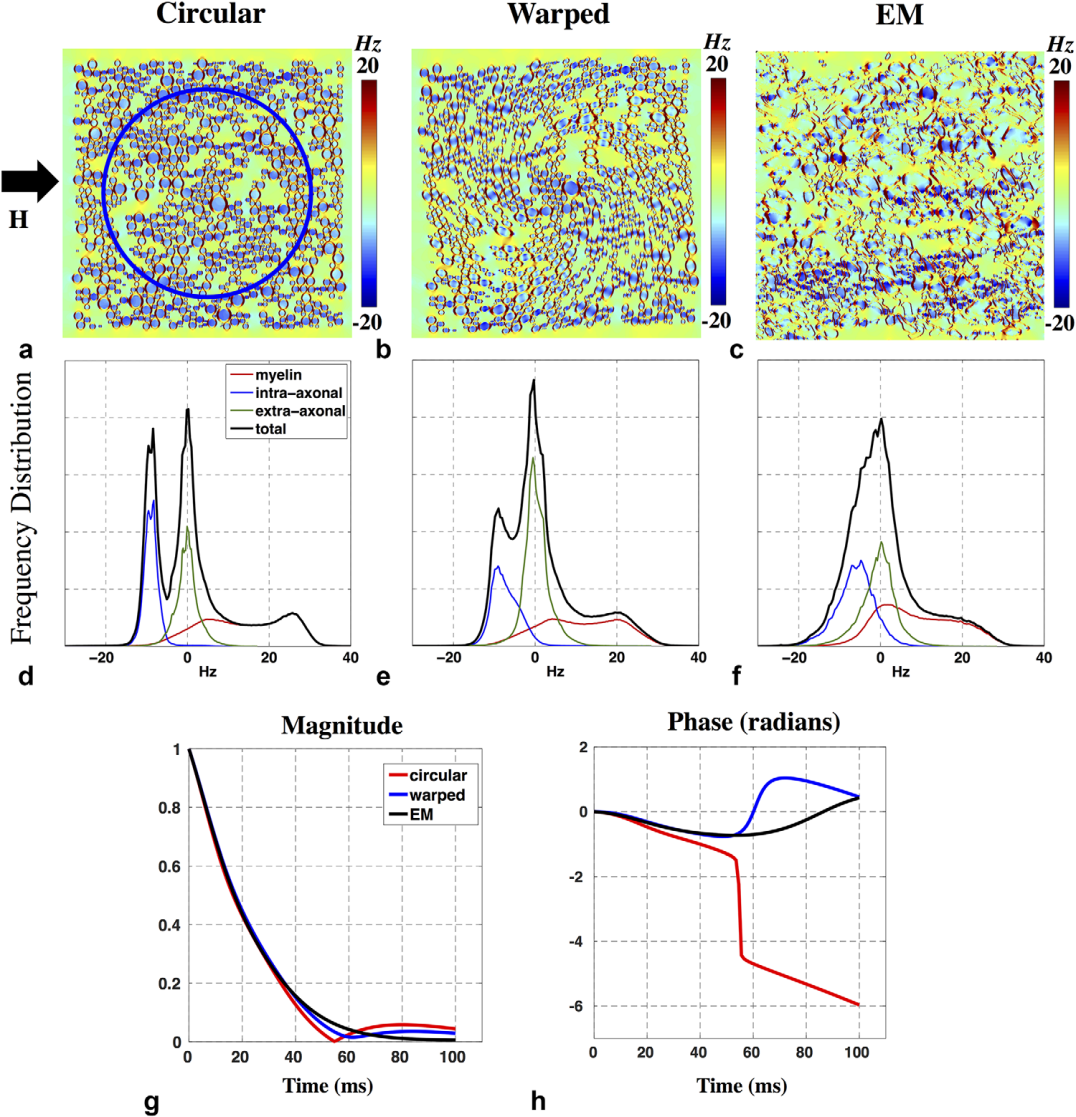
(**a**–**c**) Field perturbations corresponding to circular (a), warped (b), and hyperrealistic (c) axon geometries. Simulations correspond to 7T field strength, with axons orthogonal to the applied field. Axons were assumed to be infinite longitudinally. (**d**–**f**) Corresponding frequency distributions from each simulation. Frequency distributions from the intra‐axonal, extra‐axonal, and myelin compartments are shown in blue, green, and red, respectively. For circular axons, the distinct peak characteristics of the myelin and intra‐axonal compartments are visible in the overall distribution. In contrast, the distributions associated with warped and realistic axons are more aggregated with less distinguishable peaks. (**g, h**) Comparison of the predicted signal magnitude (g) and phase (h) across the three geometric models (circular axons, red; warped axons, blue; and EM‐derived axons, black).

The complex MR signal was computed from the field maps, ΔHz(*r,ϕ*). The total signal is a summation over the frequencies in the intra‐/extra‐axonal and myelin compartments:

(2) $$S\left(t\right)=\sum_{n=1}^{3}\rho_{n^{}}e^{-t/T_{2,n}}\int \int e^{-i2\pi t\Delta \text{Hz}\left(r,\phi \right)n}rdrd\phi$$

where *n* denotes the three compartments, for which proton density *ρ* and T_2_ were based on literature values (Table 1). Microstructure simulations model the signal decay associated with $1/T_{2}^{'}$ to arrive at the total signal decay $1/T_{2}^{*}\;=\;1/T_{2}+\;1/T_{2}^{'}$. Signal is sampled from central regions of the field maps for single axons (transparent regions in Fig. 3a–f) and axon bundles (blue circle in Fig. 5a) to avoid edge artifacts. Further details regarding artifacts and model validation are provided in Supporting Figures S4 and S5.

**Table 1:** Compartmental Properties

Compartment	T_2_ (ms)	Proton Density (*ρ*)	*χ*_isotropic_ (ppb)	*χ*_anisotropic_ (ppb)
Intra‐axonal	50	1	0	0
Extra‐axonal	50	1	0	0
Myelin	15	1/2	−60	−120

Isotropic and anisotropic magnetic susceptibility values were based on model estimates in the study by Wharton and Bowtell 14. A proton density value of 0.5 was based on the known water content of different WM compartments 43. T_2_ values for intra‐axonal, extra‐axonal, and myelin water were based on the study by Peters et al. 44, which is in fair agreement with estimates from recent work 4, 14.

Signal simulations for a multiecho GRE acquisition (e.g., free induction decay) were performed at echo times from 3 to 55 ms to match the cuprizone imaging experiment (described below). In static simulations, we calculated the signal 100 times between 0 and 55 ms, or every 0.55 ms. The plots in Figure 5g,h were simulated to 100 ms to demonstrate some of the effects at longer echo time (TE), such as the beating pattern starting at 55 ms, which is the result of distinct frequency groups in the circular model (red). In diffusion‐weighted signal simulations, the signal was calculated after each diffusion step. The signal was calculated over 550,000 intervals for the EM model and 341,191 intervals for the circular model. The number of intervals is dictated by the input step‐time, as explained in the earlier section on diffusion simulations. Furthermore, there was a wrapping of the signal phase (of 2π rad) at 55 ms in the red curve. The curve that is plotted shows the unwrapped signal phase. The sudden accrual of phase is a feature of the interference at 55 ms, where the signal magnitude temporarily reaches 0.

Calculations of the field perturbations based on 2D input susceptibility maps with array size 4000 × 4000 takes 15 seconds in MATLAB (2015b; MathWorks, Natick, Massachusetts, USA) using 8GB RAM. Monte Carlo simulations of diffusion took 3 hours using parallel computation on a multi‐node cluster (400 simulations with 250 spins each). Signal accumulation of all spins takes an additional 3 seconds.

### Electron Microscopy Acquisition

All experiments were compliant with the local regulatory and ethical standards regarding animal research. One healthy wild‐type mouse was anesthetized and perfused with normal Ringer's solution (Electron Microscopy Sciences, Hartfield, Pennsylvania, USA) and 2% formaldehyde 31. A cerebellar WM region with circular cross‐sectional areas was selected where axons are most perpendicular to the sectioning plane with orientation indicated using an ink marker. The EM image was acquired at 7.1 nm on a 4000 × 4000 matrix. Myelinated axons and myelin sheaths were hand‐segmented. Axon size, fiber density (assuming uniformity in the third dimension) and g‐ratio were calculated. Axon radius was calculated as the square root of the area of the axon divided by π. The EM image and distribution of axon sizes are shown in Supporting Figure S6.

### Cuprizone Experiment

#### Acquisition

**Figure 6:**
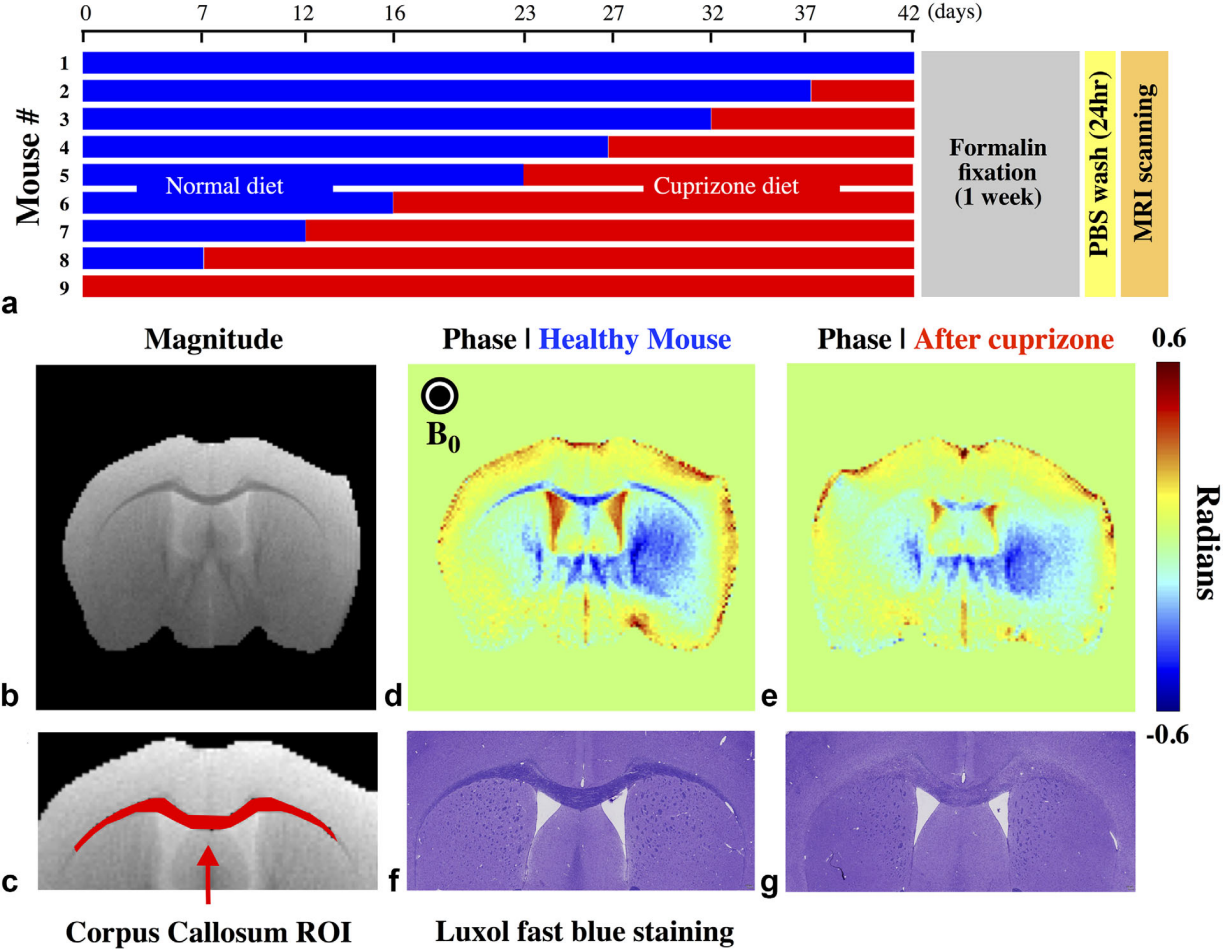
Effect of cuprizone on mouse WM. (**a**) Feeding schedule for nine mice over a 42‐day period, followed by sacrifice, fixation, and scanning. (**b**) Axial magnitude image of a mouse with no cuprizone diet. (**c**) Region of interest mask over the CC tract, which was used to collect the time‐dependent MR signal. (**d**) Phase image from a healthy mouse at TE = 23 ms. (**e**) Phase image from the mouse fed a cuprizone diet for 37 days (compared at the same TE = 23 ms). (**f, g**) Luxol fast blue histological staining for myelin for the same mice shown in panels d and e; healthy myelination is marked by high intensity stain in panel f in contrast to lower intensity staining or reduced myelin in panel g. Phase wrapping was removed using FSL PRELUDE. Background fields present in the phase images were estimated using the projection‐onto‐dipole‐fields or PDF method in 2D and then removed.

Demyelination was studied using a cuprizone mouse model in which ingestion of cuprizone, a copper chelator, leads to oligodendrocyte death and subsequent reversible demyelineation 32, 33. C57B1/6 mice (n = 9; 8 weeks old) were fed 0.2% cuprizone ad libitum for variable durations over a 42‐day period (Fig. 6a) to induce varying degrees of demyelination. Mice were sacrificed after 42 days and imaged. Supporting Table S1 lists an approximate correlation of days on the cuprizone diet to g‐ratio and volume fraction of myelin in WM.

Cuprizone mice were scanned ex vivo on a 7T preclinical scanner (Bruker Clinscan, Ettlingen, Germany) using four‐channel receive and body transmit coils. Imaging used a multiecho GRE sequence (TE = 3–55 ms, 4 ms echo spacing, TR = 1500 ms, flip angle 70*°*, FOV 10 × 10 mm, matrix 124 × 124, slice thickness 0.3 mm, 10 averages). Three axial slices were acquired at 0, 4, and 8 mm rostral to the Bregma.

#### Analysis

A region of interest of the corpus callosum (CC) was manually defined for each mouse using the magnitude image at the first TE. The raw complex GRE signal includes phase wraps and a large spatially varying background field. The background field correction was based on the phase images from the first five TEs. Spatial phase wrapping was removed 34 and background fields were estimated using 2D projection‐onto‐dipole‐fields 35. From the resulting background field estimates at the first five TEs, we calculated a voxel‐wise linear fit to the phase across TEs (ϕ(TE) = TE*m + b) to extract the component of the phase due to the background field. This linear fitting provides a correction for the raw, complex data in each voxel over all TEs: exp(*i*(TE*m + *b*)).

Recent work has suggested that the mean (nonmicroscopic) susceptibility difference between WM and gray matter (GM) also drives nonlocal field contributions 14. Nonlocal field perturbations due to WM–GM tissue interfaces were calculated with the aid of diffusion tensor imaging data, where the latter was used to account for susceptibility anisotropy based on fiber orientation 14. Calculations of the nonlocal perturbations and details of the DTI acquisition are provided in Supporting Figure S7.

#### Model comparisons

Simulations assuming only isotropic susceptibility were performed to investigate whether isotropic susceptibility was sufficient in modeling the MR signal. Field perturbations of circular and EM models (Fig. 4a,c) assuming *χ*_a_ = 0 and *χ*_i_ = −100 ppb were calculated using Equation [1]. The complex MR signal was calculated to 55 ms using Equation [2] assuming microstructure properties listed in Table 1.

Simulations of the MR signal using the circular model (Fig. 4a) under a different *χ*_a_ was performed. Field perturbations were calculated using Equation [1] assuming *χ*_a_ of −70 ppb (as opposed to −120 ppb) and *χ*_i_ to −60 ppb. Field perturbations under nine different g‐ratios, ranging from 0.70 to 0.98, were performed. The complex MR signal corresponding to each simulation was calculated using Equation [2] with parameters listed in Table 1.

## RESULTS

### Single Axon Simulations

We first investigate the effect of geometry of a single axon on the local magnetic field perturbation. Six different geometric cases and their field perturbations were generated to examine the effect of axon shape and orientation. Figure 3a–d shows axons as ellipses of increasing eccentricities, ranging from circular (eccentricity 0) to more elliptical. Figure 3e,f demonstrates the effect of rotation of the highest eccentricity ellipse relative to **H**, ranging from orthogonal to parallel. Differences in signal behavior can be attributed solely to the changes in axon shape because volume fractions of myelin, intra‐axonal space, and extra‐axonal space are conserved.

The signal magnitude and phase for each ellipse is plotted in Figure 3g,h, demonstrating distinct magnitude and phase profiles with particularly pronounced differences in signal phase. The signal phase shows increasing accumulation in the first 30 ms as the axon becomes increasingly elliptical. Rotations of a noncircular geometry can also drive significant signal changes: for example, signal phase corresponding to Figure 3d,f are opposite in sign after 55 ms despite having identical shape.

### Simulations at the Microstructural Scale

Geometries for more realistic simulations of packed axons at are shown in Figure 4. The simulated noncircular geometry is generated by warping the circular template (Fig. 4a) in a manner that conserves g‐ratio and fiber density (Fig. 4b). The EM‐based geometry is shown in Figure 4c.

Field simulations corresponding to the geometries given in Figure 4 are shown in Figure 5a–c. For circular axons, the net frequency histogram (black) exhibits three characteristic peaks, each corresponding to a tissue compartment. The intra‐ and extra‐axonal spaces form sharp peaks at −9.6 Hz (blue) and 0 Hz (green), respectively. The myelin compartment (red) is broader with two distinctive humps at ∼0 and 25 Hz. Results from the warped template show an overall smoothing and narrowing of the frequency distribution relative to the circular geometries (Fig. 5e), with the three peaks shifted toward zero. For the EM‐based simulations, the total frequency distribution shows no distinguishable peaks, but does retain a strong asymmetric shoulder (Fig. 5f).

The effect of noncircular geometries is also reflected in the simulated MR signal. The distinct peaks observed in Figure 5d produce a beating in the signal magnitude around 55 ms (Fig. 5g, red). This beating is attenuated for the warped case (blue) and is extinguished for the EM‐based simulation (black) due to the less distinct frequency peaks. The narrower distributions for the warped and EM geometries generate a more slowly varying signal phase compared with the circular geometry (Fig. 5h). Over the 55‐ms duration measured in the cuprizone experiment, the phase for the warped and EM cases accumulated 0.6 rad (34 °) in contrast to the circular case, which accrued 1.5 rad (86 °).

### Cuprizone Mouse Model Experiment Results

The experimental design and example images for the cuprizone demyelination experiment are shown in Figure 5. Histological staining confirmed reduced myelin (low stain intensity) in mice with long‐duration compared to short‐duration diets (Fig. 6f,g). Example phase images (TE = 23 ms) from a healthy mouse (Fig. 6d) demonstrate markedly stronger contrast between GM and CC compared with a mouse on a 37‐day cuprizone diet (Fig. 6e). This is consistent with a reduced myelin volume fraction in the CC, rendering the region less diamagnetic. Supporting Table S1 provides an approximate correlation between days on a cuprizone diet to g‐ratio and volume fraction of myelin in WM.

**Figure 7:**
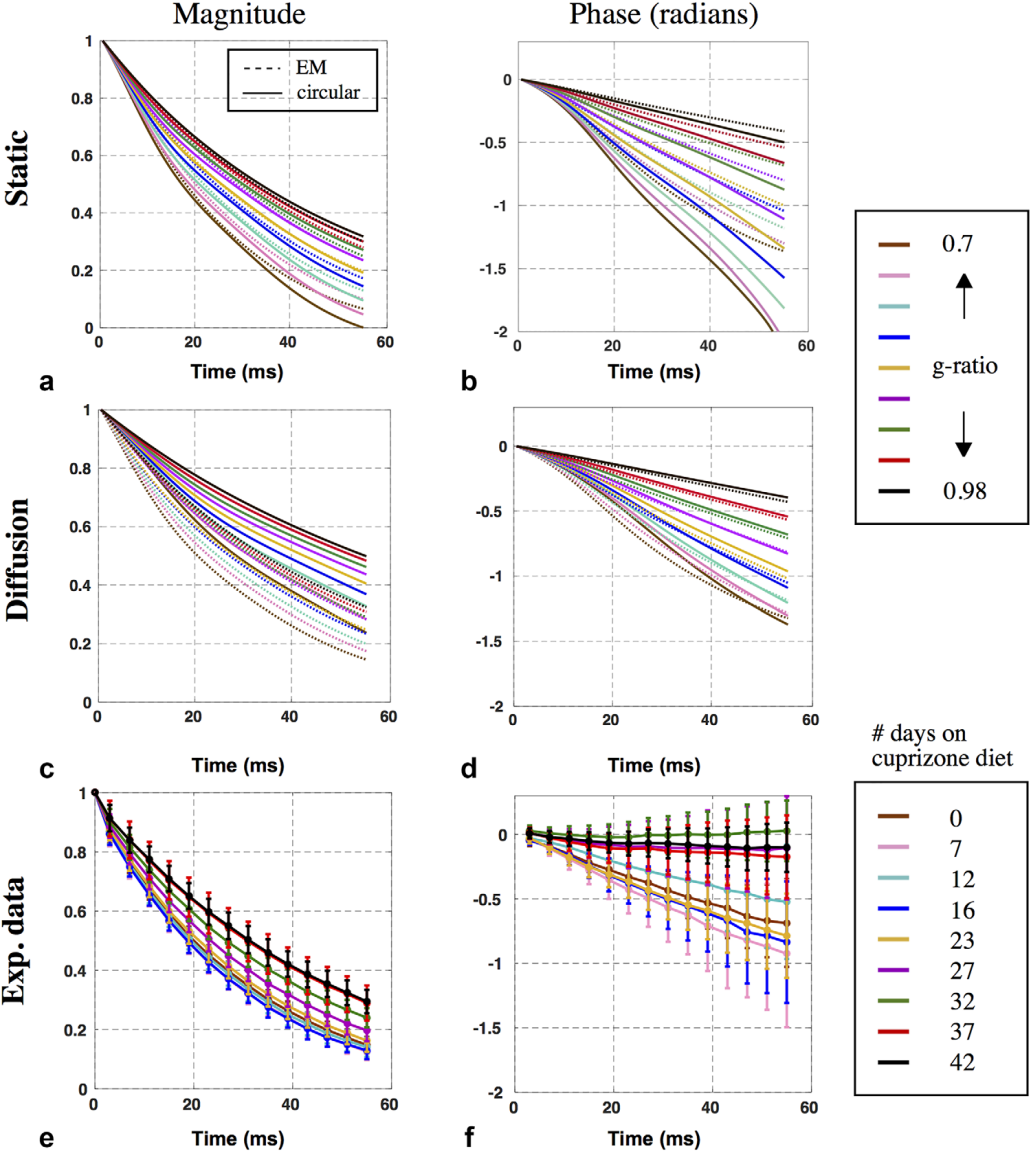
Signal modeling of demyelination compared with experimental data. (**a**–**d**) Plots of signal magnitude and phase predictions without diffusion (a, b) and with diffusion (c, d). The dotted and solid lines correspond to EM and circular models, respectively. The static magnitude predictions from the circular and EM models were 0–0.32 and 0.07–0.30 at 55 ms, respectively (a); their static phase predictions were −2.20 to −0.50 rad and −1.34 to −0.40 rad, respectively (b). The diffusion magnitude predictions from the circular and EM models were 0.23–0.49 and 0.13–0.32 at 55 ms, respectively (c); their diffusion phase predictions were −1.37 to −0.42 rad and −1.40 to −0.42 rad, respectively (d). (**e, f**) Plots of the magnitude and phase measured in the cuprizone mouse cohort.

The average signal magnitude and phase from the CC (Fig. 6c) is plotted in Figure 7e,f. The phase data have been processed to remove macroscopic field inhomogeneities (see Methods). These plots are color‐coded by the cuprizone diet duration. There is a clear trend for faster signal decay and greater phase accumulation in mice undergoing short diet durations (and therefore mostly intact myelin). At TE = 55 ms, the signal magnitude has attenuated to approximately 0.15–0.35 and the signal phase varies from −0.9 to 0 rad (−51° to 0°).

### Signal Predictions for Demyelination

Figure 7 also presents forward model predictions for the circular and EM geometries (Fig. 4a,c). The myelin sheath is eroded incrementally to simulate nine stages of demyelination wherein the g‐ratio ranges from 0.70 (healthy myelination) to 0.98 (severe demyelination). These models were calculated for both static magnetization (no diffusion; Fig. 7a,b) and diffusing magnetization (Fig. 7c,d). All models predict faster signal decay and phase evolution with higher levels of myelination. The circular model predicts somewhat greater signal decay and significantly greater phase evolution than the EM‐based model, and the shape of the signal evolution also differs between geometries. Given that these simulations were otherwise matched, these differences suggest that myelin geometry has a significant influence on both signal magnitude and phase across a range of demyelination stages. Finally, results suggest that diffusion has a significant effect on both GRE signal magnitude and phase predicted by the circular model, but considerably less effect on the EM‐based signal model. This effect is further illustrated in Supporting Figure S3.

**Figure 8:**
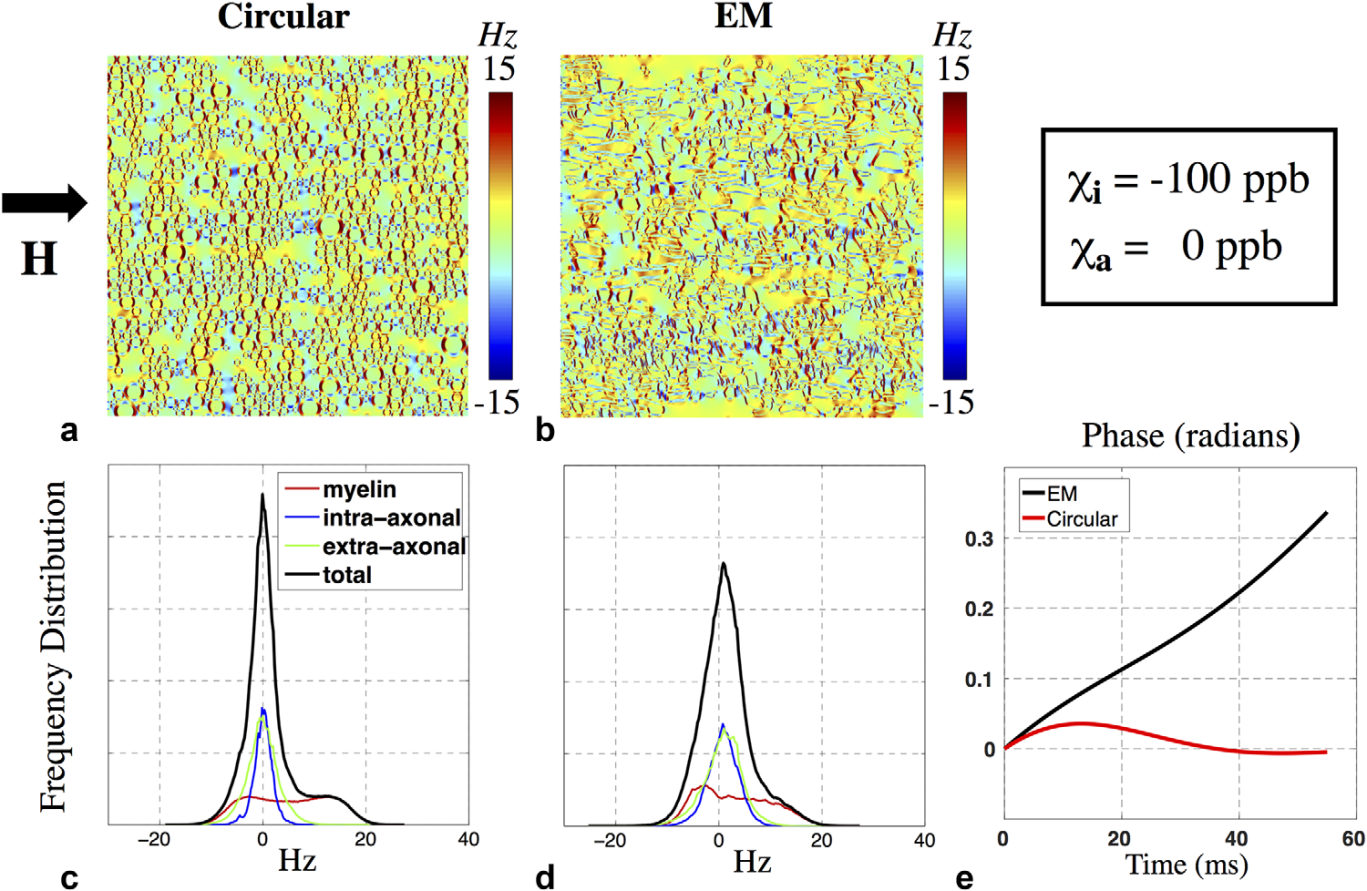
Simulations under only isotropic susceptibility, for comparison with the anisotropic model shown in Figure 5. (**a, b**) Microstructural fields generated from circular and EM geometries. (**c, d**) Corresponding frequency distributions for these two examples. The intra‐axonal frequency distribution in both models is centered about 0 Hz when only isotropic susceptibility is considered, unlike the anisotropic case. The overall distributions have a positive mean frequency due to the positive shift in the myelin compartment. (**e**) Simulations predict positive signal phase accrual under purely isotropic susceptibility, unlike the predictions shown in Figure 5.

In contrast to results where susceptibility anisotropy is included (Fig. 5), frequency distributions for purely isotropic susceptibility have a positive mean (Fig. 8c,d) and generate positive signal phase evolutions (Fig. 8e). Anisotropic susceptibility induces a negative field shift associated with the intra‐axonal compartment, resulting in negative phase evolution, consistent with experimental data.

**Figure 9:**
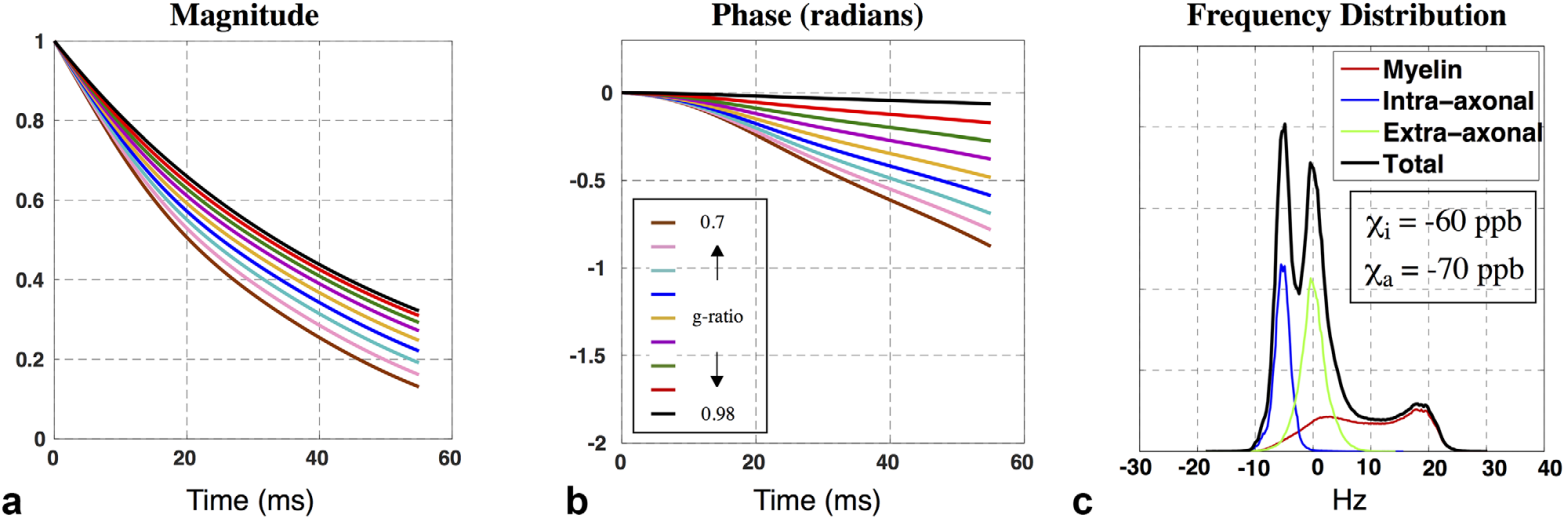
Signal predictions for circular geometries for altered susceptibility values can produce similar signal ranges to those observed experimentally. (**a, b**) The signal magnitude and phase over a range of g‐ratios with *χ*_i_ = −60 ppb and *χ*_a_ = −70 ppb. The result is similar to experimental data and to results from EM models in Figure 7c,d which assume *χ*_i_ = −60 ppb and *χ*_a_ = −120 ppb. (**c**) The change of *χ*_a_ from −120 to −70 ppb shifts the intra‐axonal frequency peak from −10 Hz (Fig. 5g) to −6 Hz, thereby slowing down the signal magnitude decay and negative phase accrual.

The signal model predictions above use literature values for key parameters such as susceptibility (*χ*_a_ = −120 ppb), which have some degree of uncertainty. Changing *χ*_a_ to −70 ppb in the circular model predicts MR signal magnitude and phase evolutions similar to experimental data (Fig. 9a,b). This single parameter change shifts the intra‐axonal frequency peak from −10 Hz (Fig. 5d) to −6 Hz (Fig. 9c) thereby slowing down the signal phase evolution. Signal simulation across the nine g‐ratios shown in Figure 9a,b does not include the nonlocal WM/GM corrections used in plots in Figure 7a–d.

## DISCUSSION

This study investigates the role of axon shape on the susceptibility‐weighted MR signal. Across geometric models, we match the WM microstructural parameters that are known to influence the MR signal (T_2_, proton density, *χ*_i_ and *χ*_a_, g‐ratio, fiber density), which allows us to attribute differences in the signal predictions to geometry. Nevertheless, other WM parameters that are not included in our simulations can also affect the MR signal, including iron‐rich oligodendrocytes, as discussed below.

Single axon simulations demonstrate that varying the eccentricity or in‐plane rotation of a myelinated axon with respect to the magnetic field alters the MR signal behavior, implying loss of specificity for biophysical properties such as g‐ratio. To probe richer geometries on a larger scale, we also considered packed axonal bundles. As with single axons, changes to the myelin shape modulate the underlying frequency distribution and the MR signal, such that a multiplicity of MR signals can be generated from packings sharing the same g‐ratio and fiber density.

### Implications of Simulations

The frequency distributions for the simulated geometries exhibit a characteristic set of peaks, which become narrower and less distinct as the simulated geometries become more realistic. These results have important implications for methods aiming to quantify myelin properties from the susceptibility‐weighted signal. For example, a logarithmic relationship has been derived between the intra‐axonal field shift and the g‐ratio for the nested cylinder geometry 14, suggesting a possible in vivo measure for g‐ratio. For our g‐ratio of 0.7, our circular axon geometry predicts an intra‐axonal field shift of −9.6 Hz (Fig. 5d) that is consistent with the analytic description. However, simulations from the EM‐based model predict that individual compartment distributions are blurred and closer to zero offset, resulting in an aggregation in the frequency distribution and a disappearance of this characteristic intra‐axonal peak (Fig. 5f). We have demonstrated that these differences introduce significant alterations to susceptibility‐weighted MR signal properties like those used to estimate g‐ratio.

Assuming that the EM model is a more accurate reflection of the true underlying microstructure, the implication of these results is two‐fold. First, attempts to extract microstructure parameters such as g‐ratio from the MR signal would need to incorporate the effect of shape. Second, these results suggest that estimates of myelin susceptibility obtained by fitting circular models of myelin geometry to the MR signal are biased. Under an identical set of parameters (including the same g‐ratio and fiber density, therefore myelin content) EM and circular models predict different MR signal. If the EM model is a more accurate representation of white matter microstructure, fitting based on the circular model could underestimate myelin content (Fig. 7b).

Our results therefore highlight a challenge for the use of susceptibility signals for estimating biophysical properties: simple models like circular geometries appear invertible but are unlikely to be accurate, while more realistic geometries like EM‐based templates are not a particularly practical approach to biophysical modeling.

### Effect of Diffusion

Simulations that include diffusion predict slower signal magnitude decay and slower signal phase accrual in both circular and EM models compared with static simulations. This is consistent with “motional narrowing,” in which spins experience less dephasing as a result of random diffusion 36. Diffusion had a larger influence on the signal predictions for the circular model than the EM model (Fig. 7c,d) and reduces the difference in signal phase predictions for the EM and circular models (Fig. 7b,d). However, the signal magnitude is predicted to be more different between circular and EM geometries when diffusion is included (Fig. 7a,c). Supporting Figure S8 provides an alternative to Figure 7, which compares diffusion and static cases directly.

Incorporating diffusion has the primary effect of reducing the more extreme signal predictions for the circular model, as are seen for the most highly myelinated cases (i.e., the solid lines in Fig. 7a,b are overall very different from the other lines of the same color in Fig. 7a–d). Previous work has suggested that incorporation of diffusion into the susceptibility‐based WM signal model can produce more accurate estimates of the experimental data 4. Our signal predictions that include diffusion (taking parameters from literature) are in good agreement with the measured signals from the mouse model of demyelination. However, the significance of this agreement should not be interpreted too strongly in light of the dependence of the signal predictions on parameters like susceptibility with some uncertainty (Fig. 9). Rather, the realism of the EM geometries and diffusion simulations provide evidence that these properties are important to accurate signal prediction.

### Limitations of the Study

Field simulations shown in Figure 5a‐c were performed in 2D assuming that all axons are parallel and infinite in the third dimension. This is an important remaining simplification regarding the structure of axons, which in reality undulate and deform along tracts. Moreover, in EM data of mouse brain WM, microstructures other than axons were observed though not included in the simulation. For example, one such structure, iron‐rich oligodendrocytes, occupy significant volume fractions in some areas of the WM and can have significant effects on the susceptibility‐weighted signal 37.

In comparing signal simulations of demyelination to the cuprizone mouse measurements, we make the implicit assumption that there is a monotonic relationship between the duration of cuprizone feeding and demyelination (see Supporting Table S1). The MR signal measurements do not demonstrate a strict monotonicity with feeding duration, but they do demonstrate the expected overall trend if one groups the mice according to short, intermediate, and long duration diets. This could in part reflect differences in the feeding behavior of different animals or differences in the neurobiological response to cuprizone.

In modeling demyelination, we made the assumption that fiber density remains unaffected as the myelin sheath is thinned from the inside out. In reality, the mechanism by which myelin clearance occurs is more complex; different stages of demyelination can be characterized by either myelin debris or clearance 38. After myelin loss, the demyelinated axon may be surrounded by enlarged astrocytic processes coupled with an increase of microglial cells 39. Increases in the population of astrocytes have also been reported, although the volume fraction of extra‐axonal space is countered by the decrease in oligodendrocytes 33, 40. Such changes in the extra‐axonal volume fraction as well as the spatial distribution of myelin throughout the demyelination process could affect the signal behavior.

Changes to axonal properties were minimized by perfusion fixation of the mouse brain. Measurements of axon size (n *=* 602; see Supporting Fig. S4) followed a gamma distribution with mean of 0.46 μm, in agreement with previous studies 41.

DTI data were used to compute the nonlocal fields from bulk WM/GM distribution. DTI to date provides the best nondestructive measure of fiber orientation available for whole brains. However, there are known shortcomings of DTI, including inaccuracies in areas with multiple fiber populations. We assume that the principal diffusion direction is aligned to the longitudinal axis of the axon and therefore the *χ*_⊥_ component of the susceptibility tensor shown in Figure 1a. Results from susceptibility tensor imaging (STI) in large fiber bundles are consistent with the principal axis in DTI; however, STI in small fiber bundles, as well as some larger fiber bundles (e.g., the superior regions of CC), has been shown to differ from DTI data in some regions 42. Future simulations of nonlocal WM/GM distortion may benefit from incorporating STI data with DTI data.

### Failure of the Isotropic‐Only Susceptibility Model

Recent studies have suggested that myelin exhibits anisotropic magnetic susceptibility and that accounting for this property can provide accurate descriptions of the MR signal modeling, particularly signal phase. Our results show that without susceptibility anisotropy, the overall frequency distribution is positive and predicts a positive phase evolution that is not observed in experimental data (Fig. 8). Incorporating susceptibility anisotropy and/or nonlocal bulk WM/GM field perturbations may produce an overall negative frequency distribution, which would in turn predict negative phase evolutions as seen in the data.

### Circular Geometries Versus EM Geometries

Parameter values used in the simulations were based on the literature (Table 1) 14. Under these specific values, results from the EM model provide greater agreement with the measured data. However, there is still considerable uncertainty about the magnetic susceptibility of myelin, which has a significant effect on the signal prediction. The results shown in Figure 9 suggest that both circular and EM models can produce MR signal behavior that agrees with the measurements. Moreover, the plots in Figure 9 did not include nonlocal WM/GM corrections. The only sense in which one can consider the EM‐based model to be better is that it is based on more realistic microstructural properties taken from EM measurements. This highlights the problematic nature of using simplified biophysical models of MRI signals to estimate MRI‐relevant tissue properties that drive those signals (e.g., myelin susceptibility) and also to estimate microstructural tissue properties of neurobiological significance (e.g., g‐ratio).

## CONCLUSIONS

The results from this study suggest that myelin geometry affects the MR signal. Signal predictions using axon models with realistic geometries, with compartmental properties from literature, were significantly different to circular models and were in good agreement with experimental data from a cuprizone‐mouse demyelination model. A powerful application of susceptibility‐weighted imaging is the potential to estimate tissue properties such as myelin magnetic susceptibility, fiber density and g‐ratio 4, 6, 14, 15. This is sought by fitting biophysical models to the measured MR signal. Our results show that these estimates are likely to be biased by assuming simplified, circular geometric models. Elliptical and EM‐based models may provide an opportunity to improve the extraction of such tissue parameters. As such, a careful and thorough understanding of the role of shape in the modulation of the MR signal is essential.

## Supporting information

Additional Supporting Information may be found in the online version of this article

**Table S1**. Predicted Correlation Between Days Spent on a Cuprizone Diet, g‐Ratio, and Volume Fraction *v* of Myelin in White Matter
**Fig. S1**. (**a**) Random close packing of n = 1434 circles within a square area 37 × 37 μm^2^. Packing fiber density reaches 83%. (**b**) Circle radii follow a gamma distribution with a mean of 0.46 μm, based on literature values.
**Fig. S2**. Orientation of myelin phospholipid to the magnetic field in the azimuth plane for a single segmented axon taken from EM data.
**Fig. S3**. The effect of diffusion is compared for (**a**) EM and (**b**) circular models. Unmyelinated axons are packed into the extracellular space for more a realistic representation of WM. These models have an extra‐axonal volume fraction of 25%, in contrast to the volume fraction of 36% associated with the models in Figure 4. (**c, d**) Plots of the static and diffusion‐weighted signal magnitude and phase. The results demonstrate that diffusion has a more significant effect on the circular geometry in both signal magnitude and phase. However, unmyelinated axons had little effect on the signal magnitude and phase. As such, we adopted a myelinated‐axon model (Fig. 4) throughout this study for both static and diffusion‐weighted simulations.
**Fig. S4**. (**a**) Single axon field perturbation calculated using the Fourier method, described by Equation [1], assuming the magnetic field is perpendicular to the longitudinal axis of the axon. (**b**) Single axon perturbation generated by plotting the analytic solutions or ground truth. (**c**) Difference between ground truth and Fourier method results at a color bar windowing of −30 to 30 Hz. (**d**) Plot of the difference rewindowed to −2 to 2 Hz emphasizes edge artifacts from Fourier transform operations and the segmentation of the myelin sheath into quadrilaterals. Outer edge artifacts are avoided by sampling within a central field of view (black circle). Segmentation‐induced artifacts are not avoided. (**e, f**) Comparison of the signal magnitude and phase calculated from field perturbations in (a) and (b) with the central field of view. The results suggest that the segmentation‐based Fourier method is a good approximation of the analytic solutions.
**Fig. S5**. (**a, b**) Plots of the signal magnitude and phase from six separate simulations. The number of axons in these six simulations ranges from 1434 to 52 and is color‐coded. In each simulation a central field of view, which samples 50% of the simulation area, was used to extract the frequencies for signal calculation. (**c, d**) Comparison of the signal magnitude and phase between a circular (n *=* 1434) and EM (n *=* 52) model where the size of the FOV is varied. The number of axons sampled within the FOV changes and is color‐coded. The black solid line (labeled C′) represents the case where 600 circular axons are simulated and 300 axons are sampled. These simulations suggest that the shape of axons influences the MR signal more than the number of axons simulated as well as the number of axons sampled.
**Fig. S6**. (**a**) EM image of mouse cerebellar WM, matrix size = 4000 × 4000 acquired at a resolution of 7.1 nm. (**b**) Histogram of axon radii size with Gamma fit yielding shape factor, *α* = 5.7 and mean radius of 0.46 μm.
**Fig. S7**. (**a**) Mean offset in the CC region of interest produced by the nonlocal WM/GM perturbations as a function of myelin volume fraction, *v*, in WM. *v* ranges from 0.32 (healthy) to 0.03 (demyelinated). (**b, c**) Signal phase predictions from circle model without and with nonlocal correction, respectively. (**d, e**) Signal phase predictions from EM model without and with nonlocal correction, respectively.
**Fig. S8**. Signal modeling of demyelination compared with experimental data. (**a**–**d**) Plots compare signal magnitude and phase predictions across circular (a, b) and EM (c, d) models. Dotted and solid lines correspond to diffusion and static results, respectively. (**e, f**) Plots show the magnitude and phase measured in the cuprizone mouse cohort.
