# Supplementary material for: The effect of realistic geometries on the susceptibility‐weighted MR signal in white matter
Source: Magn Reson Med. 2017 Apr 10;79(1):489–500. doi: 10.1002/mrm.26689 (PMC6585669; doi:10.1002/mrm.26689)
Supplement: Supplementary file 1 — Table S1. Predicted Correlation Between Days Spent on a Cuprizone Diet, g‐Ratio, and Volume Fraction v of Myelin in White Matter Fig. S1. (a) Random close packing of n = 1434 circles within a square area 37 × 37 μm2. Packing fiber density reaches 83%. (b) Circle radii follow a gamma distribution with a mean of 0.46 μm, based on literature values. Fig. S2. Orientation of myelin phospholipid to the magnetic field in the azimuth plane for a single segmented axon taken from EM data. Fig. S3. The effect of diffusion is compared for (a) EM and (b) circular models. Unmyelinated axons are packed into the extracellular space for more a realistic representation of WM. These models have an extra‐axonal volume fraction of 25%, in contrast to the volume fraction of 36% associated with the models in Figure 4. (c, d) Plots of the static and diffusion‐weighted signal magnitude and phase. The results demonstrate that diffusion has a more significant effect on the circular geometry in both signal magnitude and phase. However, unmyelinated axons had little effect on the signal magnitude and phase. As such, we adopted a myelinated‐axon model (Fig. 4) throughout this study for both static and diffusion‐weighted simulations. Fig. S4. (a) Single axon field perturbation calculated using the Fourier method, described by Equation [1], assuming the magnetic field is perpendicular to the longitudinal axis of the axon. (b) Single axon perturbation generated by plotting the analytic solutions or ground truth. (c) Difference between ground truth and Fourier method results at a color bar windowing of −30 to 30 Hz. (d) Plot of the difference rewindowed to −2 to 2 Hz emphasizes edge artifacts from Fourier transform operations and the segmentation of the myelin sheath into quadrilaterals. Outer edge artifacts are avoided by sampling within a central field of view (black circle). Segmentation‐induced artifacts are not avoided. (e, f) Comparison of the signal magnitude and phase calculated from field p [file MRM-79-489-s001.docx]

**Supporting Material**

**Theory: field perturbation calculations**

The total field perturbation originating from myelin is the sum of the field contributions from isotropic susceptibility **χ_i_,** where tensor components *χ*_‖_ = *χ*_⊥_$,$and anisotropic susceptibility **χ_a_**, where *χ*_‖_ ≠ *χ*_⊥_$.$ These tensors, **χ_i_** and **χ_a_**, in their non-rotated frames are shown in Figures 2b and 2c. The magnitude of **χ_a_** and **χ_i_** used in our calculations are based on estimations in previous work (Table 1) (1).

Orientation of *χ*_‖_ to the applied field **H** is plotted for two geometric scenarios: orthogonal cross-sections of a “nested cylinder” and “nested elliptical cylinder,” shown by the shapes in Figures 2d and 2e. For the isotropic case, where *χ*_‖_ = *χ*_⊥_, the susceptibility tensor in the rotated frame (Fig. 1b) becomes an identity matrix. This simplifies the calculation of the frequency field in Equation 1 to the well-known convolution of the susceptibility distribution with a dipole kernel (2). In contrast, field calculations for the anisotropic case rely on explicit information on the orientation of *χ*_‖_ to **H**. This is shown by the color-coded maps in Figures 2d and 2e, spanning 0—2π. The field perturbations corresponding to **χ_i_** and **χ_a_** are generated in Figures 2f and 2g for the circular geometry and in 2h and 2i for the elliptical geometry. Note that the result in Figure 2f represents the canonical model of cylindrical axons considering only isotropic magnetic susceptibility. In this work, calculations were performed in 2D assuming axon geometries were constant in the third dimension. The orientation of magnetic field, *θ,* is assumed to be orthogonal to the longitudinal axis of the axon(s).

**Circle packing**

First, circles (*n=*1434) are generated following a Gamma distribution (α=5.7) with a mean of 0.46 μm. A circle is selected at random without replacement. The placement or position of the selected circle is determined such that its distance to the average center of all the circles already placed is minimized without overlap. This process is iterated until all circles are placed. The algorithm is uploaded and available here (3). The packing reaches a fiber density of ~83% and is then modified to reach a myelinated fiber density of 64% by random removal of circles and to have an average g-ratio of 0.71. These parameters (density=64%, mean axon size=0.46 μm, g-ratio= 0.71) match the measurements taken from the EM dataset and are also in agreement with literature (4,5). The fiber density measured in the EM data represents only myelinated axons, the population relevant to modeling myelin susceptibility, and is thus lower than would be expected for both myelinated and unmyelinated axons. Results of circle packing is shown in Figure S1.

**Myelin sheath segmentation**

First, the myelin sheath is outlined along its inner circumference and outer circumference. These outlines are then divided (*n=*200) into equally spaced segments. Starting with one point on the inner circumference, a line is made to the nearest point on the outer circumference. Next, a connection is made to the subsequent point (still on the outer circumference). Then, a line is made to a point back on the inner circumference that is one segment away from the original starting point. Finally, the two points on the inner circumference are joined, forming a closed quadrilateral. We assume that the phospholipids traverse the shortest path between the layers of the myelin sheath. The results are shown in Supporting Figure S2. The EM dataset from mouse cerebellar WM is shown in Figure S4.

**Model validation and signal simulation**

Central regions were sampled in single axons simulations for two reasons. The first is due to edge artifacts from discrete Fourier transform operations. A comparison between the analytic solutions, which is assumed to be ground truth, and the Fourier method is shown in the Supporting Figure S4 highlighting greater inconsistencies near the edges. The second reason is that sampling a central region keeps the volume fraction (assuming continuity in the 3^rd^ dimension) to a more reasonable value. The extra-axonal volume fraction without restricting sampling to the circular FOV would be ~87%. This value is unrealistic for white matter microstructure. Incorporating a FOV reduces the extra-axonal volume fraction to 37% (Figures 3a-f). This is still a high volume fraction according to some literature (6,7). However, this FOV allows for the more eccentric ellipses (Figures 3d-f) to fit within the FOV. The EM model contains fewer axons than the circular bundle model (*n*=602 vs 1434). However, g-ratio and fiber density between the circle and EM models are matched. The FOV is scaled such that total spatial area sampled is consistent across the two models.

We also examined whether the number of axons simulated had an impact on the signal predictions as well whether varying the size of FOV for sampling (and therefore the number of axons) affected signal predictions. Figures S8a and S8b plot the signal magnitude and phase from six separate simulations. The number of axons in these six simulations is color coded, ranging from 1434 to 52. In each simulation a central FOV, which samples 50% of the simulation area, was used to extract the frequencies for signal calculation. Therefore signal predictions come from sampling ~700 to ~25 axons. Results indicate that changes in axon quantity have a minimal impact on signal magnitude and phase compared to a change in axon geometry (Figs. 4g and 4h). In general, simulating a larger number of axons would produce greater accuracy for the model, as it increases homogeneity. Our choice for a model with ~1400 axons was a balance between computation resources, time and accuracy.

Next, we examined whether the size of the sampling FOV affected signal predictions. This was achieved by varying the sizes of the FOV mask on the model of 1434 circular axons and 602 EM axons. Figures S8c and S8d show the magnitude and phase under different FOVs for the circular model (solid lines) and EM model (dashed lines). These lines are color coded to the number of axons sampled within the FOV. The solid black line (C’) represents the case where 600 axons are simulated and 300 axons are sampled by the FOV. The results suggest that the size of the central FOV has a negligible effect on the signal magnitude and phase in the circular model. In contrast, we see larger differences in signal phase for the EM case as we vary the FOV size. This is likely because axons in our EM dataset are not as uniformly and homogeneously distributed as is in our circular simulations (Fig. 4c). In the future, it may benefit to use larger EM datasets (>30 μm). There is little variation in signal magnitude for the EM model as FOV is varied. Cross comparison between the circular and EM model results indicate that axonal shape drives the signal changes much more strongly than the number of axons sampled.

**Diffusion simulations**

For the susceptibility model presented here, unmyelinated axons can be neglected when calculating field perturbations under the assumption that their magnetic susceptibility is matched to the extra-axonal space. However, unmyelinated axons exist in abundance and will impede diffusion as their membranes represent boundaries, and thus may have an impact on the calculated signal. To examine whether the inclusion of unmyelinated axons is necessary for model accuracy, we performed additional simulations that packed circular unmyelinated axons with a mean radius of 0.2 μm and standard deviation of 0.05 μm into the extra-axonal space in Figures 4a and 4c to produce Figures S3a and S3b, respectively. Unmyelinated axons have a significantly smaller radius than myelinated axons (8). Diffusion was simulated separately for each of the four compartments (extra-axonal, myelin, intra-axonal, unmyelinated axons) assuming impermeable membranes, and the resulting signal was compared to our standard model without unmyelinated axons. Our results demonstrate that the presence of unmyelinated axons had almost no effect on the signal magnitude or phase (Supporting Figs. S3c and S3d). As such, we use a simpler geometry that does not include unmyelinated axons throughout this paper (Figs. 4a and 4c) for both static and diffusion-weighted simulations.

**Nonlocal field perturbations from WM and GM**

A WM mask was generated by applying a threshold to the fractional anisotropy data (FA>0.25). Next, the principal diffusion direction in each voxel in the WM mask was used to determine the orientation of the principal axis of the susceptibility tensor relative to the applied field, analogous to mapping of the orientation of *χ*_||_ to **H** in Figures 2d and 2e. Nonlocal field perturbations were forward calculated in 3D. Experimental measurements from the cuprizone mouse are in 2D, of axial slices through the corpus callosum. We created ROIs of the corpus callosum within the 3D simulation, in axial slices that were anatomically matched as closely as possible to the experimental data. The average field offset *F*(*Hz*) was measured from these ROIs and was added to the signal *S(t)* calculated in Equation 2, which represents the contribution from only the local microstructure. The corrected signal had the form: *S_c_(t)* = *S(t)* exp(*i*2π*Ft*), where *F(Hz)* was the average field offset. Signal predictions in Figures 7a-d include nonlocal contributions. Calculations of nonlocal WM/GM contributions and microstructure contributions both assume literature values (1,8,9). Their effect on the MR signal is shown in Supporting Figure S7.

Field distortions generated from WM/GM susceptibility contrast is a weighted summation of the isotropic and anisotropic susceptibility field contributions, shown in Equation A1. This equation follows Equation S25 in (14),

 [A1]

where *v* is the volume fraction of myelin in WM, *χ_i_ ,χ_f_ ,χ_a_* are the scalar isotropic susceptibility value of myelin relative to its surroundings, scalar isotropic susceptibility of WM relative to GM and scalar anisotropic susceptibility of myelin, respectively, and ΔHz_i_ and ΔHz_a_ are the field perturbations arising from the isotropic susceptibility (Fig. 2b) and anisotropic susceptibility (Fig. 2c), which are calculated using Equation 1. In these nonlocal simulations *χ*_i_ =-60 ppb, *χ*_f_=-20 ppb and *χ*_a_ =-120 ppb. Volume fraction of myelin in WM or *v* is calculated as *v* = (1-$g^{2}$)$\cdot$ $d$ where $g$ is the g-ratio and $d$ is density of axons. In our simulations $d=$ 63%. As g-ratio varies from 0.70 to 0.98, *v* decreases from 0.32 to 0.03, shown in Table S1.

The magnitude of the nonlocal field is a function of *v*. As such, nine simulations were performed with *v* ranging from 0.32 (healthy) to 0.03 (demyelinated). Simulations were performed in 3D. We created ROIs of the corpus callosum within the 3D simulation in axial slices that were matched anatomically as closely as possible to the experimental data. The ROIs were made using the fractional anisotropy map (FA>0.25). The average field offset is obtained from these ROIs and is shown in Figure S5a, ranging from -1.72 to -0.96 *Hz*. This offset was added to the complex signal, described by Equation 2, arising from microstructure fields (Figs. 4a and 5c) shown in Figure S5b and S5c. For example, the offset from the simulation with *v*=0.32 was added to the signal corresponding to the microstructure simulation where the g-ratio is 0.70. The effect of the nonlocal fields is significant at long echo times. In the EM model, the signal phase from healthy WM (green curve) accrues 0.75 radians in 55 ms without the nonlocal field addition, Figure S5c. The effect of the correction (offset of -1.72*Hz*) causes the signal phase to evolve more rapidly to -1.4 radians in the same time, a nearly 200% change, in Figure S4c.

**Field-of-view in single axon simulation**

Figure S6a shows the field perturbation arising from a single axon (of g-ratio 0.6) generated using the Fourier method described by Equation 1. Figure S6b plots the field perturbations from by their analytic solutions (14). The analytic solutions are assumed to be the ground truth. The difference, by subtraction, between the two fields is shown in Figure S6c, viewed through a colorbar window of -30 to 30 *Hz.* In Figure S6d, this windowing is changed to -2 to 2 *Hz* to emphasize the edge artifacts which result from the discrete Fourier transforms used in Equation 1. Further, the effect of quadrilateral segmentation of the myelin sheath is accentuated. A circular and central FOV is used avoid sampling the edges of this square array where differences in field are pronounced. Figure S6e and S6f compares the MR signal magnitude and phase arising from the fields in Figure S6a and S6b. The results suggest that the Fourier method offers a fair approximation to the analytic solutions.

**Supporting Table S1.** Predicted Correlation Between Days Spent on a Cuprizone Diet, g-Ratio, and Volume Fraction *v* of Myelin in White Matter

**
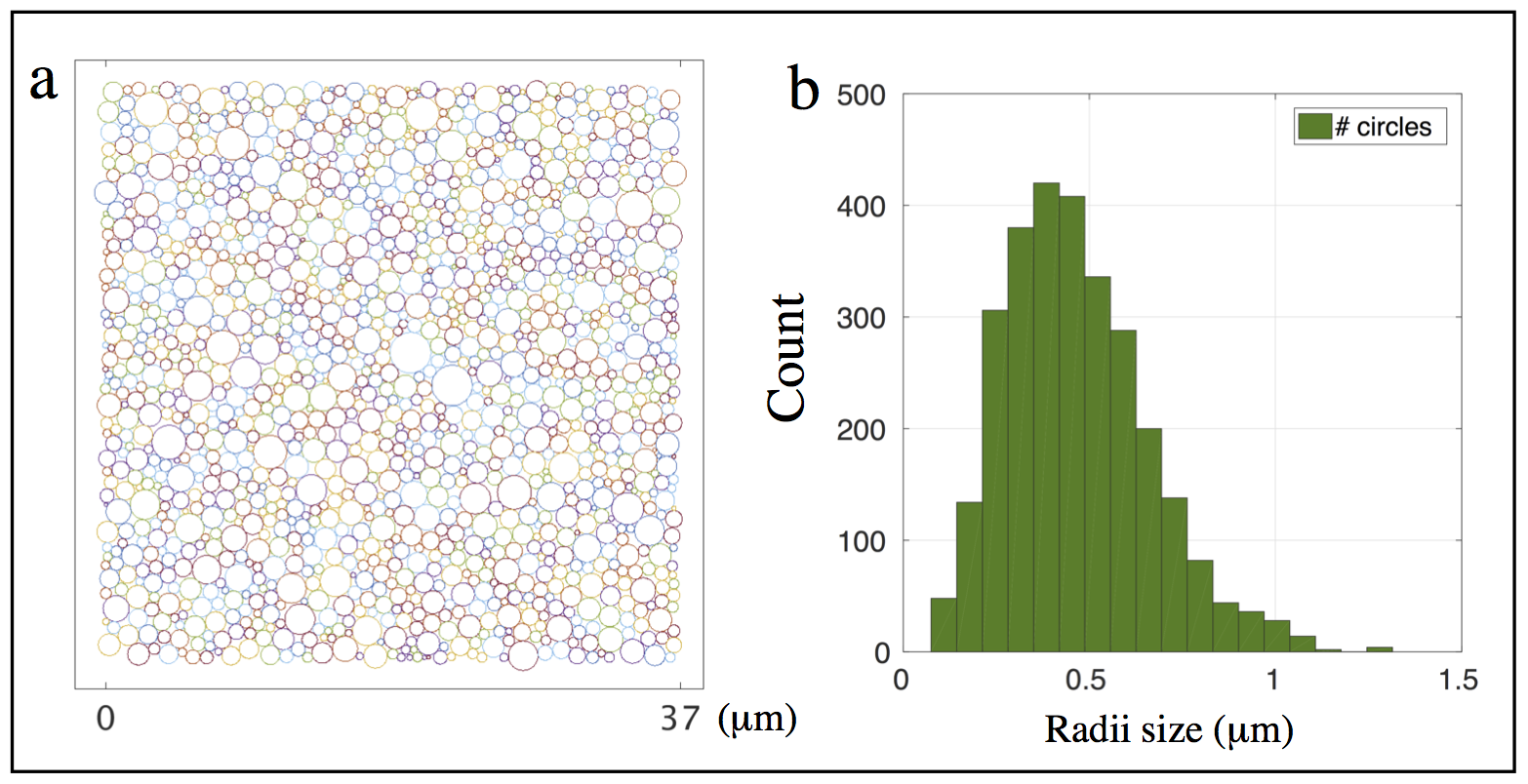
**

**Supporting Fig. S1.** (**a**) Random close packing of n = 1434 circles within a square area 37 × 37 μm^2^. Packing fiber density reaches 83%. (**b**) Circle radii follow a gamma distribution with a mean of 0.46 μm, based on literature values.

**
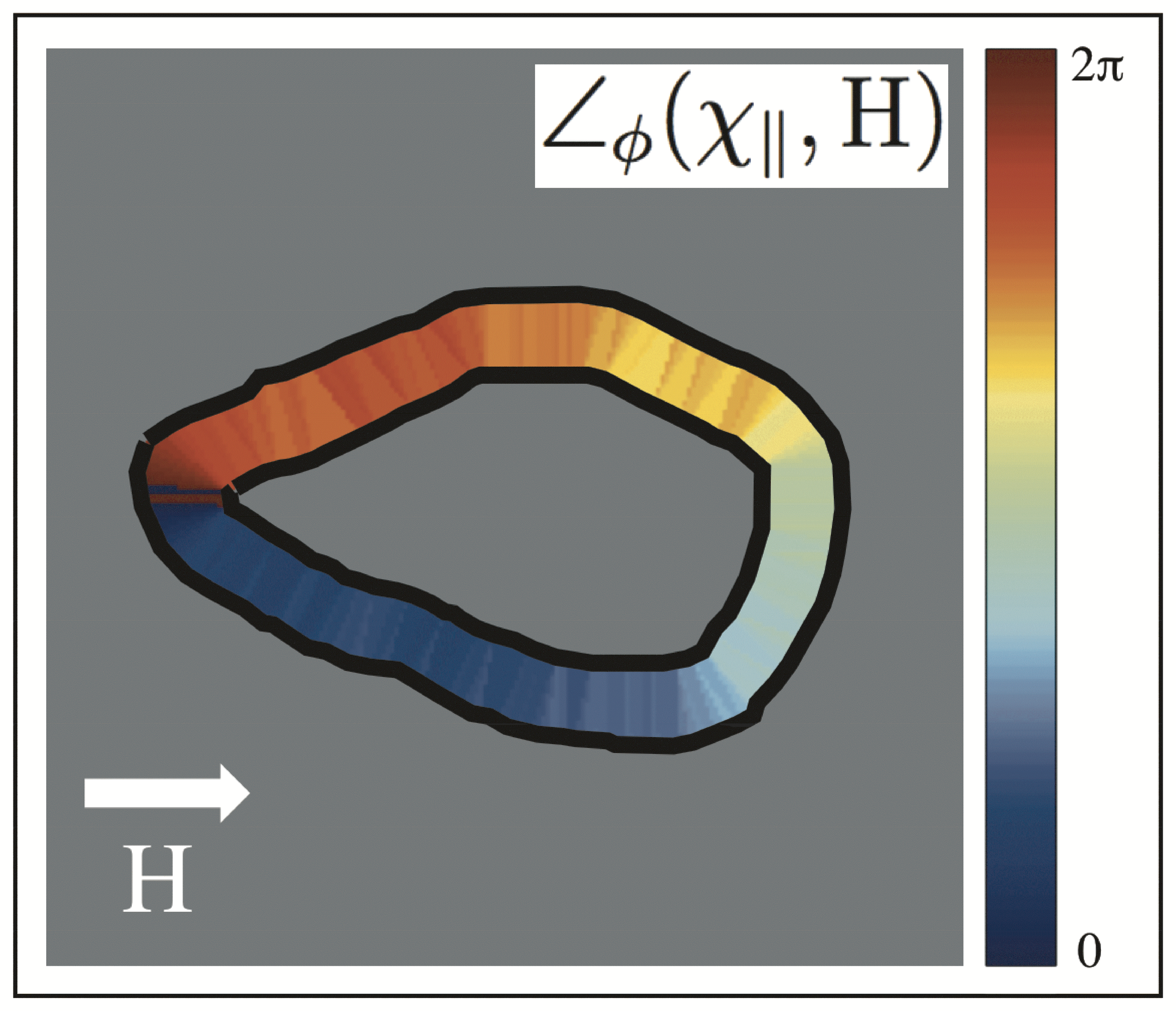
**

**Supporting Fig. S2.** Orientation of myelin phospholipid to the magnetic field in the azimuth plane for a single segmented axon taken from EM data.


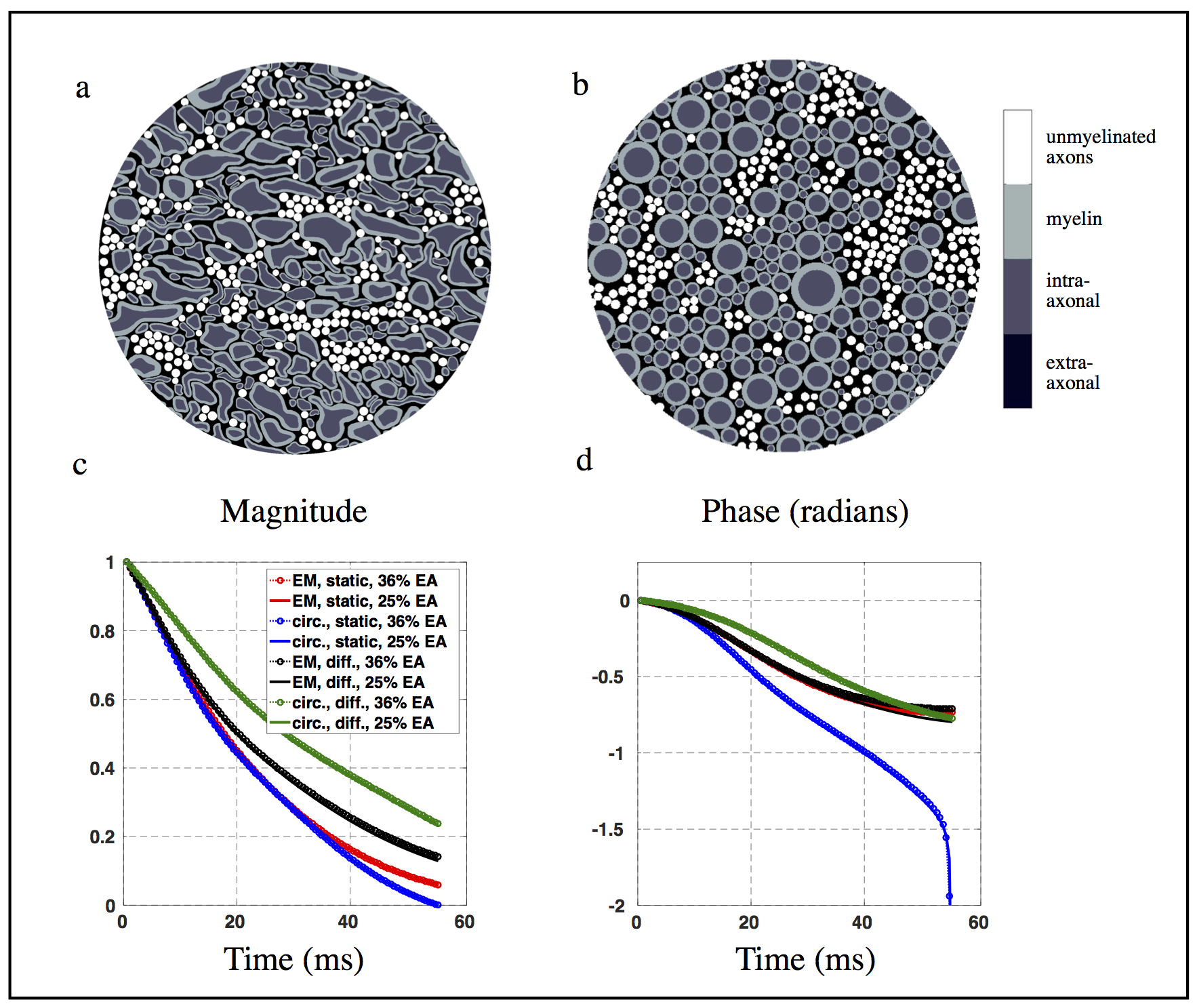


**Supporting Fig. S3.** The effect of diffusion is compared for (**a**) EM and (**b**) circular models. Unmyelinated axons are packed into the extracellular space for more a realistic representation of WM. These models have an extra-axonal volume fraction of 25%, in contrast to the volume fraction of 36% associated with the models in Figure 4. (**c**, **d**) Plots of the static and diffusion-weighted signal magnitude and phase. The results demonstrate that diffusion has a more significant effect on the circular geometry in both signal magnitude and phase. However, unmyelinated axons had little effect on the signal magnitude and phase. As such, we adopted a myelinated-axon model (Fig. 4) throughout this study for both static and diffusion-weighted simulations.


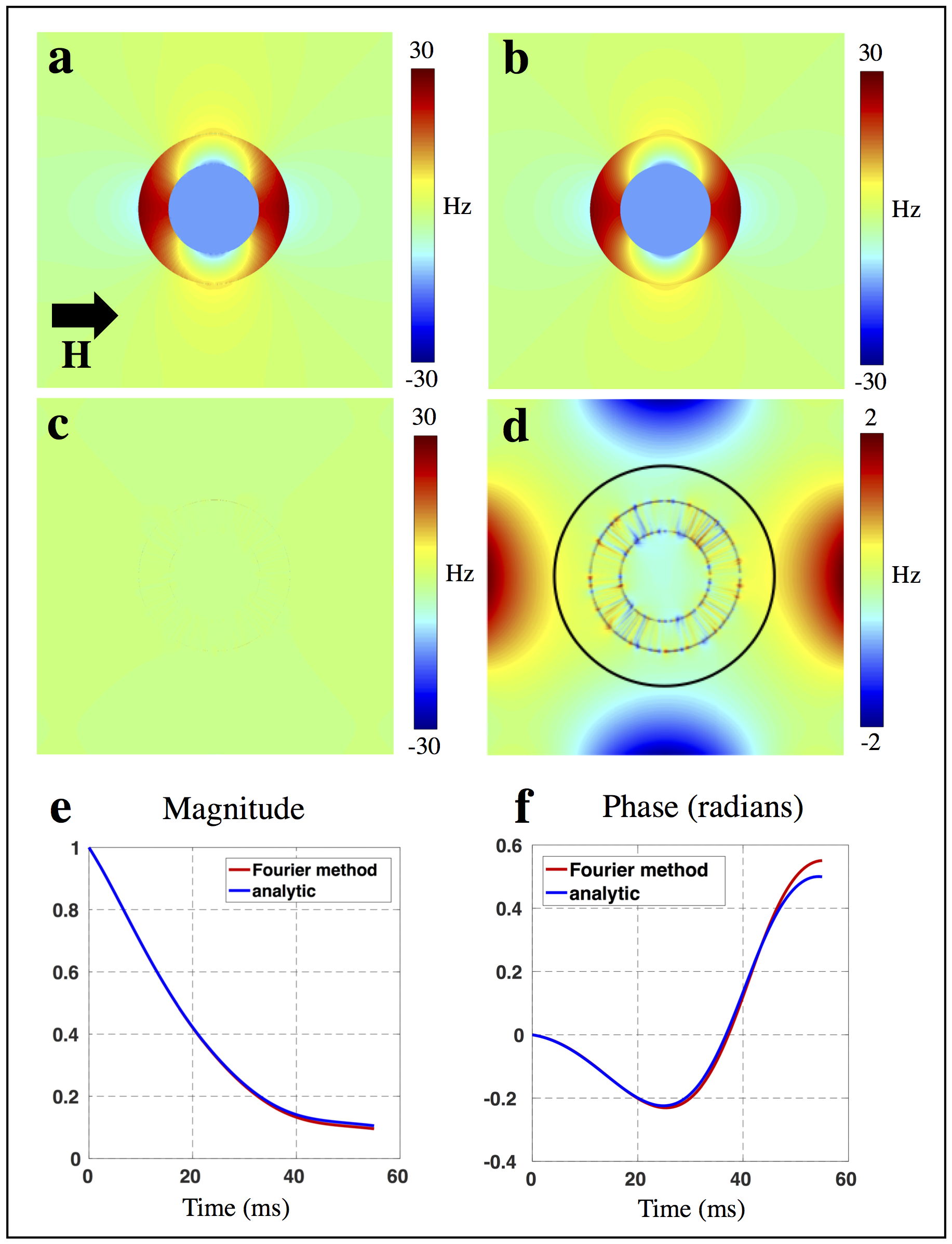


**Supporting Fig. S4.** (**a**) Single axon field perturbation calculated using the Fourier method, described by Equation 1, assuming the magnetic field is perpendicular to the longitudinal axis of the axon. (**b**) Single axon perturbation generated by plotting the analytic solutions or ground truth. (**c**) Difference between ground truth and Fourier method results at a color bar windowing of −30 to 30 Hz. (**d**) Plot of the difference rewindowed to −2 to 2 Hz emphasizes edge artifacts from Fourier transform operations and the segmentation of the myelin sheath into quadrilaterals. Outer edge artifacts are avoided by sampling within a central field of view (black circle). Segmentation-induced artifacts are not avoided. (**e**, **f**) Comparison of the signal magnitude and phase calculated from field perturbations in (a) and (b) with the central field of view. The results suggest that the segmentation-based Fourier method is a good approximation of the analytic solutions.


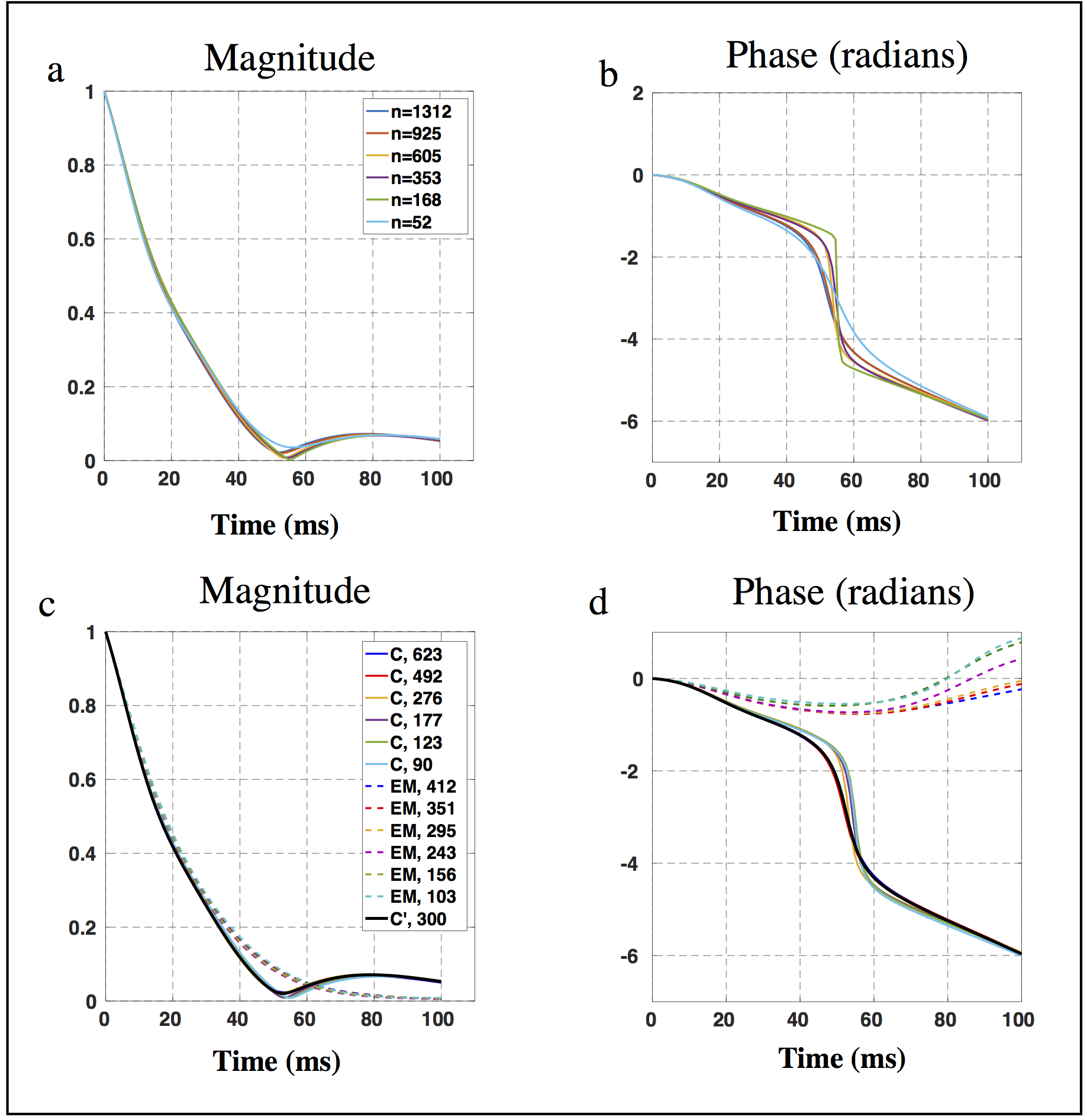


**Supporting Fig. S5.** (**a**, **b**) Plots of the signal magnitude and phase from six separate simulations. The number of axons in these six simulations ranges from 1434 to 52 and is color-coded. In each simulation a central field of view, which samples 50% of the simulation area, was used to extract the frequencies for signal calculation. (**c**, **d**) Comparison of the signal magnitude and phase between a circular (n *=* 1434) and EM (n *=* 52) model where the size of the FOV is varied. The number of axons sampled within the FOV changes and is color-coded. The black solid line (labeled C’) represents the case where 600 circular axons are simulated and 300 axons are sampled. These simulations suggest that the shape of axons influences the MR signal more than the number of axons simulated as well as the number of axons sampled.


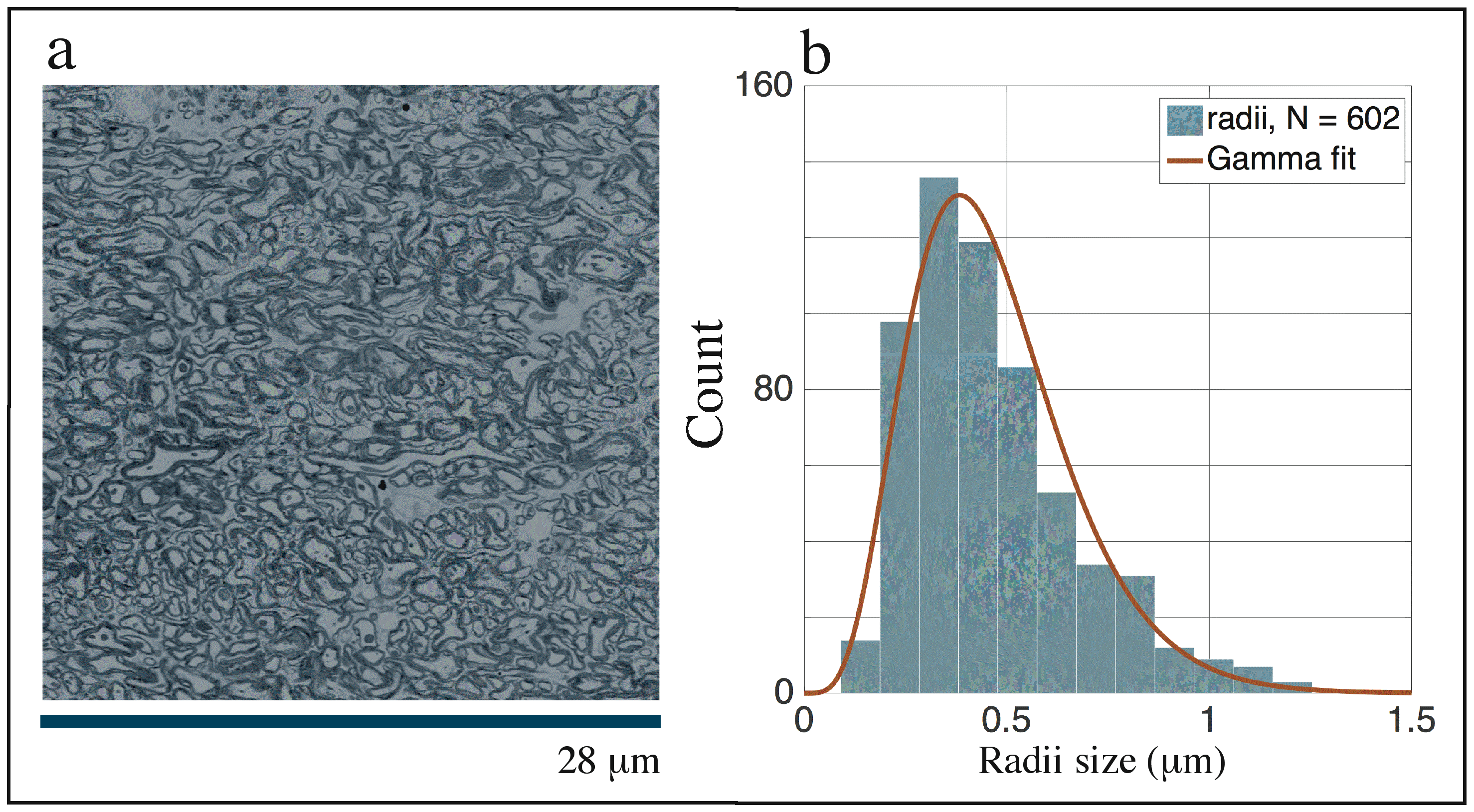


**Supporting Fig. S6.** (**a**) EM image of mouse cerebellar WM, matrix size = 4000 × 4000 acquired at a resolution of 7.1 nm*.* (**b**) Histogram of axon radii size with Gamma fit yielding shape factor, *α* = 5.7 and mean radius of 0.46 μm.


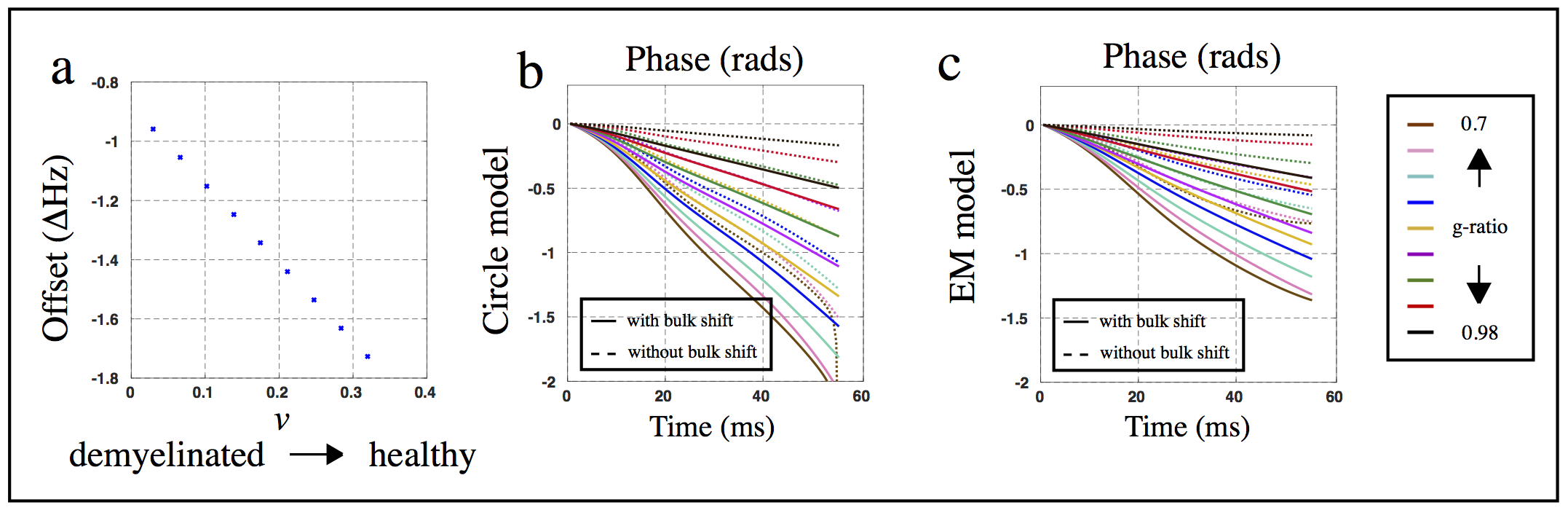


**Supporting Fig. S7.** (**a**) Mean offset in the CC region of interest produced by the nonlocal WM/GM perturbations as a function of myelin volume fraction, *v*, in WM. *v* ranges from 0.32 (healthy) to 0.03 (demyelinated). (**b**, **c**) Signal phase predictions from circle model without and with nonlocal correction, respectively. (**d**, **e**) Signal phase predictions from EM model without and with nonlocal correction, respectively.


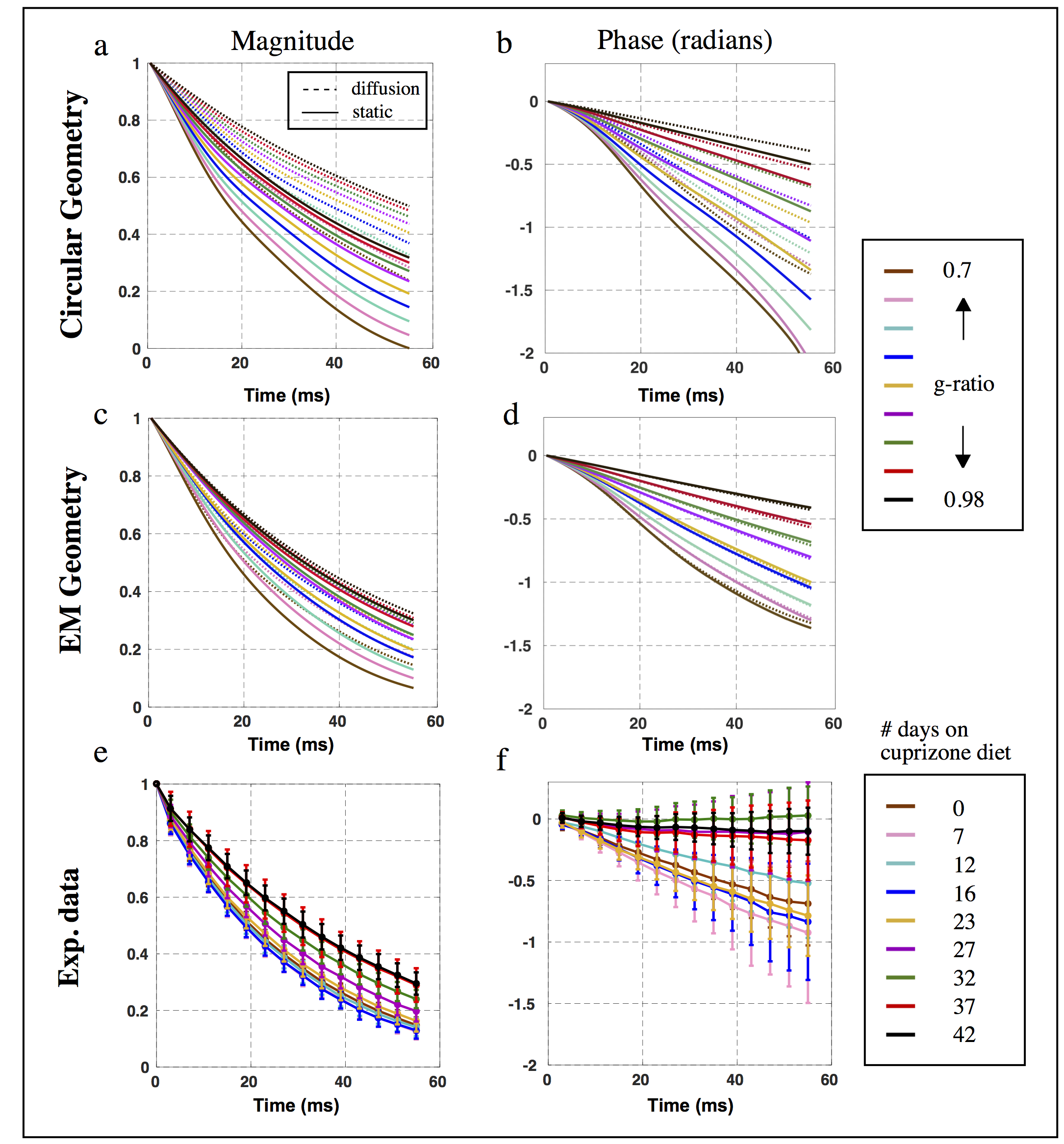


**Supporting Fig. S8.** Signal modeling of demyelination compared with experimental data. (**a**–**d**) Plots compare signal magnitude and phase predictions across circular (a, b) and EM (c, d) models. Dotted and solid lines correspond to diffusion and static results, respectively. (**e**, **f**) Plots show the magnitude and phase measured in the cuprizone mouse cohort.
